# RAD51 protects against nonconservative DNA double-strand break repair through a nonenzymatic function

**DOI:** 10.1093/nar/gkac073

**Published:** 2022-02-08

**Authors:** Ayeong So, Elodie Dardillac, Ali Muhammad, Catherine Chailleux, Laura Sesma-Sanz, Sandrine Ragu, Eric Le Cam, Yvan Canitrot, Jean Yves Masson, Pauline Dupaigne, Bernard S Lopez, Josée Guirouilh-Barbat

**Affiliations:** Université de Paris, INSERM U1016, UMR 8104 CNRS, Institut Cochin, Equipe Labellisée Ligue Contre le Cancer, France; CNRS UMR 8200, Gustave-Roussy, Université Paris-Saclay, 114 rue Edouard Vaillant, 94805 Villejuif, France; Université de Paris, INSERM U1016, UMR 8104 CNRS, Institut Cochin, Equipe Labellisée Ligue Contre le Cancer, France; CNRS UMR 8200, Gustave-Roussy, Université Paris-Saclay, 114 rue Edouard Vaillant, 94805 Villejuif, France; Genome Maintenance and Molecular Microscopy UMR 9019 CNRS, Université Paris-Saclay, Gustave Roussy, F-94805, Villejuif Cedex, France; CBI, CNRS UMR5088, LBCMCP, Toulouse University, Toulouse, France; Genome Stability Laboratory, CHU de Québec Research Center (Oncology Division), Quebec City, QC, Canada; Department of Molecular Biology, Medical Biochemistry and Pathology, Laval University Cancer Research Center, Quebec City, QC, Canada; Université de Paris, INSERM U1016, UMR 8104 CNRS, Institut Cochin, Equipe Labellisée Ligue Contre le Cancer, France; CNRS UMR 8200, Gustave-Roussy, Université Paris-Saclay, 114 rue Edouard Vaillant, 94805 Villejuif, France; Genome Maintenance and Molecular Microscopy UMR 9019 CNRS, Université Paris-Saclay, Gustave Roussy, F-94805, Villejuif Cedex, France; CBI, CNRS UMR5088, LBCMCP, Toulouse University, Toulouse, France; Genome Stability Laboratory, CHU de Québec Research Center (Oncology Division), Quebec City, QC, Canada; Department of Molecular Biology, Medical Biochemistry and Pathology, Laval University Cancer Research Center, Quebec City, QC, Canada; Genome Maintenance and Molecular Microscopy UMR 9019 CNRS, Université Paris-Saclay, Gustave Roussy, F-94805, Villejuif Cedex, France; Université de Paris, INSERM U1016, UMR 8104 CNRS, Institut Cochin, Equipe Labellisée Ligue Contre le Cancer, France; CNRS UMR 8200, Gustave-Roussy, Université Paris-Saclay, 114 rue Edouard Vaillant, 94805 Villejuif, France; Université de Paris, INSERM U1016, UMR 8104 CNRS, Institut Cochin, Equipe Labellisée Ligue Contre le Cancer, France; CNRS UMR 8200, Gustave-Roussy, Université Paris-Saclay, 114 rue Edouard Vaillant, 94805 Villejuif, France

## Abstract

Selection of the appropriate DNA double-strand break (DSB) repair pathway is decisive for genetic stability. It is proposed to act according to two steps: 1-canonical nonhomologous end-joining (C-NHEJ) *versus* resection that generates single-stranded DNA (ssDNA) stretches; 2-on ssDNA, gene conversion (GC) *versus* nonconservative single-strand annealing (SSA) or alternative end-joining (A-EJ). Here, we addressed the mechanisms by which RAD51 regulates this second step, preventing nonconservative repair in human cells. Silencing RAD51 or BRCA2 stimulated both SSA and A-EJ, but not C-NHEJ, validating the two-step model. Three different RAD51 dominant-negative forms (DN-RAD51s) repressed GC and stimulated SSA/A-EJ. However, a fourth DN-RAD51 repressed SSA/A-EJ, although it efficiently represses GC. In living cells, the three DN-RAD51s that stimulate SSA/A-EJ failed to load efficiently onto damaged chromatin and inhibited the binding of endogenous RAD51, while the fourth DN-RAD51, which inhibits SSA/A-EJ, efficiently loads on damaged chromatin. Therefore, the binding of RAD51 to DNA, rather than its ability to promote GC, is required for SSA/A-EJ inhibition by RAD51. We showed that RAD51 did not limit resection of endonuclease-induced DSBs, but prevented spontaneous and RAD52-induced annealing of complementary ssDNA *in vitro*. Therefore, RAD51 controls the selection of the DSB repair pathway, protecting genome integrity from nonconservative DSB repair through ssDNA occupancy, independently of the promotion of CG.

## INTRODUCTION

Genetic instability is a hallmark of aging and cancer (1–3). DNA double-strand breaks (DSBs) are highly toxic lesions and an important source of genome rearrangements and mutagenesis. However, DSBs are also used by the cell to generate beneficial genetic diversity in processes such as meiosis, establishment of the immune repertoire, and neuronal gene expression (for a review, see (4)). Therefore, DSB repair should be tightly controlled to maintain genome stability while allowing diversity, and the choice of the appropriate DSB repair mechanism is a crucial issue for the maintenance of genome stability.

Cells employ two primary strategies to repair DSBs: the first strategy relies on sequence homology with an intact DNA partner and thus refers to homologous recombination (HR), including conservative gene conversion (GC) associated or not with crossing over. GC is (i) initiated by resection of the DSB producing 3′-single-stranded DNA (ssDNA); (ii) the loading of RAD51 onto the ssDNA by BRCA2, which generates an ordered ssDNA/RAD51 filament that promotes the search for the homologous partner and strand invasion; (iii) DNA synthesis primed on the invading 3′-end and (iv) resolution of the intermediate leading to GC with or without crossing over. The second DSB repair strategy, nonhomologous end-joining (NHEJ), joins and ligates two DNA double-strand ends without requiring sequence homology; in canonical NHEJ (C-NHEJ), the Ku70/Ku80 heterodimer binds to the DSB and recruits DNA-PKcs, Ligase 4 and its cofactors (4–9). Both GC and C-NHEJ are essential for genome stability maintenance, but other mechanisms that are exclusively mutagenic/nonconservative can also repair DSBs. Single-strand annealing (SSA) is also initiated by resection, but instead of strand exchange with a homologous duplex DNA, two revealed long complementary ssDNA sequences anneal in a RAD51-independent but RAD52-dependent manner (10–12). In addition, another end-joining (EJ) process, A-EJ (alternative end-joining), also known as alt-NHEJ, B-NHEJ (back-up nonhomologous end-joining) or MMEJ (microhomology-mediated end-joining), has been revealed in the absence of KU or XRCC4/Ligase 4 in mammalian cells (4–8,13–15). Similarly to GC and SSA, A-EJ is initiated by ssDNA resection controlled by MRE11/CtIP (13,16,17); joining of the two resected double-strand ends is then mediated by annealing of microhomologies (few bp). Importantly, both SSA and A-EJ are nonconservative processes that inevitably lead to deletions of the intervening sequence (7,18) or translocations (19).

Because HR, SSA and A-EJ are all initiated by resection, in opposition to C-NHEJ, we have proposed that, instead of direct competition of NHEJ *versus* HR, selection of the DSB repair mechanism occurs in 2 steps (7,13): 1- competition between C-NHEJ *versus* resection; 2- on resected DNA ends, competition between GC, A-EJ and SSA. Consistent with this model, studies in rodent cells have shown that defects in C-NHEJ stimulate GC and A-EJ (5,6,20,21), and depletion of 53BP1, a key factor in DNA end protection that prevents resection, alleviates HR defects in BRCA1-deficient human cells (22). Moreover, inhibition of HR through expression of dominant negative forms of RAD51 or deficiency in BRCA2 leads to the stimulation of SSA or A-EJ (11,23–30).

While the regulation of the first step of the repair pathway choice is well documented, the molecular mechanisms governing the second step, i.e., the selection between GC *versus* SSA or A-EJ, remains poorly explored, and little is known about the mechanisms that protect genome stability against nonconservative SSA and A-EJ. One hypothesis suggests that HR prevents nonconservative SSA and A-EJ solely by engaging DSB repair toward GC. Alternatively, one can hypothesize that the presence of RAD51 on resected ssDNA might protect against essential steps (resection and/or annealing) of SSA and A-EJ independently of its ability to promote GC. Indeed, RAD51 has been shown to protect arrested replication forks from extensive resection (31–33).

Here, we address the molecular mechanisms by which RAD51 controls the choice of the DSB repair process at the second step of DSB repair pathway selection, i.e. GC *versus* SSA or A-EJ, in human cells. We performed, in parallel, a systematic analysis of different DSB repair pathways, GC, SSA and EJ, comparing the impact of siRNA and dominant-negative forms of RAD51 (DN-RAD51s) that differ in their DNA binding capacities. We found that all DN-RAD51s with altered ATPase activity failed to bind damaged DNA in living cells and led to an increase in SSA. Another DN-RAD51 that harms GC but retains the capacity to bind damaged chromatin, repressed SSA and A-EJ, unveiling a nonenzymatic role of RAD51, independent of its GC activity. This protective role of RAD51 acted through inhibition of the annealing of complementary ssDNA rather than protection against extended resection. Collectively, our data reveal an additional molecular mechanism by which RAD51 maintains genome stability in human cells: the repression of ssDNA annealing through ssDNA occupancy impairs nonconservative repair. This process is separable from the ability of RAD51 to promote GC.

## MATERIALS AND METHODS

### Cells

We used cell lines with intrachromosomal substrates to monitor GC, SSA, and EJ (both C-NHEJ and A-EJ) after targeted induction of a DNA double strand break by meganuclease I-SceI. The RG37 cell line (34) was derived from SV40-transformed GM639 human fibroblasts and stably contained pDR-GFP, a GC reporter, which restores a functional GFP gene upon I-SceI cleavage (35). GC92 cells (13) are also derived from SV40-transformed GM639 human fibroblasts and contain the pCD4-3200bp substrate that monitors EJ by expression of the membrane antigen CD4. Because I-SceI cleaves in two noncoding sequences, both error-prone and error-free repair are measured, *i.e*., both C-NHEJ (conservative repair) and A-EJ (exclusively mutagenic repair) are recorded. The sequence of the repair junction allows the estimation of the C-NHEJ/A-EJ ratio (5,6,13,36). The DIvA cell line (Asi-SI-ER-U2OS) is a U2OS cell line (human osteosarcoma) that was previously established and described by Iacovoni *et al.* (2010). DNA double strand breaks are induced at specific regions in the genome by the Asi-SI endonuclease. Asi-SI is sequestered in the cytoplasm, and after the addition of 4-hydroxy-tamoxifen (300 nM) to the culture medium for 4 h, Asi-SI translocates into the nucleus and cuts DNA. All cells were cultured in DMEM supplemented with 10% fetal calf serum (FCS) and 2 mM glutamine and were incubated at 37°C with 5% CO_2_. U2OS-SSA (37,38), U2OS EJ2-GFP (A-EJ reporter) and U2OS EJ5-GFP (C-NHEJ reporter) were kindly provided by Dr. Jeremy Stark (City of Hope, CA, USA) and are described in (38). U2OS DR-GFP was constructed in the laboratory by introducing the pDR-GFP reporter (35) into U2OS cells and is described in (39). All U2OS cells were maintained in DMEM supplemented with 10% FCS.

### Transfection

Meganuclease I-SceI was expressed by transient transfection of the pCMV-HA-I-SceI expression plasmid (40) with Jet-PEI according to the manufacturer's instructions (Polyplus transfection, New York, NY, USA, #101-40N). The expression of HA-tagged I-SceI was verified by western blotting under each condition. For silencing experiments, 25,000 cells were seeded 1 day before transfection; these experiments were performed using INTERFERin following the manufacturer's instructions (Polyplus Transfection, New York, NY, USA, #409-50) with 20 nM of one of the following siRNAs: Control (5′-AUGAACGUGAAUUGCUCAA-3′), RAD51-1 (Dharmacon, Lafayette, CO, USA, cat# L003530-00-0010), RAD51-2 (5′-GAAGCUAUGUUCGCCAUUA-3′), RAD51-3 (3′-UTR, 5′GACUGCCAGGAUAAAGCUU-3′), RAD51-4 (3′UTR , 5′-GUGCUGCAGCCUAAUGAGA-3′), BRCA2 (5′-GCUGAUCUUCAUGUCAUAA-3′) or RAD52 (5′-CCAACGCACAACAGGAAAC-3′), all of which except those ordered from Dharmacon were synthesized by Eurofins (Ebersberg, Germany). Seventy-two hours later, the cells were transfected with the pCMV-HA-I-SceI expression plasmid.

### Measurement of gene conversion, SSA and EJ efficiency by FACS

After transfection with the I-SceI expression plasmid and incubation for 72 h, the cells were collected with 50 mM EDTA diluted in PBS, pelleted and fixed with 2% paraformaldehyde for 20 minutes. The percentage of GFP-expressing cells was scored by FACS analysis using a BD Accuri C6 flow cytometer (BD, Franklin Lakes, NJ, USA). The percentage of CD4-expressing cells was measured after incubation for 10 minutes with 1 μl of anti-CD4 antibody coupled to Alexa 647 (rat isotype, RM4-5, Invitrogen, Waltham, MA, USA). For each cell line, at least 3 independent experiments were performed, and HA-I-SceI expression and silencing efficiency were verified each time by western blot analysis.

### Western blotting

For Western blot analysis, the cells were lysed in buffer containing 20 mM Tris–HCl (pH 7.5), 1 mM Na_2_EDTA, 1 mM EGTA, 150 mM NaCl, 1% (w/v) NP40, 1% sodium deoxycholate, 2.5 sodium pyrophosphate, 1 mM β-glycerophosphate, 1 mM NA_3_VO_4_ and 1 μg/ml leupeptin supplemented with cOmplete ULTRA Tablets (Roche, Basel, Switzerland). Denatured proteins (20–40 μg) were electrophoresed in 9% SDS-PAGE gels or MiniPROTEAN® TGX™ 4–15% Precast gels (Bio-Rad, Hercules, CA, USA) or on 3–8% Tris-acetate gels (Thermo Fisher Scientific, Waltham, MA, USA) transferred onto a nitrocellulose membrane and probed with specific antibodies, including anti-Vinculin (1/8000, SPM227, ab18058, Abcam, Cambridge, UK), anti-RAD51 (1/1000, Ab-1, PC130, Millipore, Burlington, MS, USA), anti-RAD52 (1/500, sc-365341, Santa Cruz, Dallas TX, USA), anti BRCA2 (1/500, OP95, Millipore, Burlington, MS, USA), anti-PALB2 (1/1000, GTX85263, Genetex, Irvine, CA, USA) and anti-HA (1/1000, HA.11 clone 16B12, Covance, Princeton, NJ, USA). Immunoreactivity was visualized using an enhanced chemiluminescence detection kit (ECL, Pierce).

### Junction sequence analysis

We amplified the junction sequences through PCR of genomic DNA using the CMV-6 (5′-TGGTGATGCGGTTTTGGC-3′) and CD4-int (5′-GCTGCCCCAGAATCTTCCTCT-3′) primers. The predicted size of the PCR product was 732 nt. The PCR products were cloned with a TOPO PCR cloning kit (Invitrogen Life Technologies, Waltham, MA, USA) and sequenced (Eurofins, Ebersberg, Germany). For each sample, two to five independent experiments were analyzed. In each experiment, HA-I-SceI expression and the silencing efficiency were verified by Western blotting.

### Immunofluorescence

Cells were seeded onto slides and transfected with empty vector, Flag-SMRAD51, Flag-WTRAD51, Flag-RAD51 K133A and Flag-RAD51 K133R, HA-WTRAD51 or HA-RAD51-T131P. Forty-eight hours after transfection, the cells were washed with PBS, treated with CSK buffer (100 mM NaCl, 300 mM sucrose, 3 mM MgCl_2_, 10 mM Pipes pH 6.8, 1 mM EGTA, 0.2× Triton and protease inhibitor cocktail (Complete ULTRA Tablets, Roche, Basel, Switzerland) and fixed in 2% paraformaldehyde for 15 min. The cells were then permeabilized in 0.5% Triton-X 100 for 10 min, saturated with 2% BSA and 0.05% Tween 20 and probed with anti-Flag (1/400, F3165 Sigma-Aldrich, St Louis, MO, USA), anti-HA (1/100, sc-7392, Santa Cruz, Dallas TX, USA), or anti-RAD51 (1/500, PC130, Millipore, Burlington, MS, USA) antibodies for 2 h at RT or overnight at 4°C. After three washes in PBS-Tween 20 (0.05%) at RT, the cells were probed with Alexa-coupled anti-mouse or anti-rabbit secondary antibody (1/1000, Invitrogen Life technologies, Waltham, MA, USA) for 1 h at RT. After three washes, the cells were mounted in DAKO (Agilent, Santa Clara, CA, USA) mounting medium containing 300 μM DAPI and visualized using a fluorescence microscope (Zeiss Axio Observer Z1) equipped with an ORCA-ER camera (Hamamatsu). Image processing and focus counting were performed using ImageJ software.

### Coimmunoprecipitation

GC92 cells grown in 75 cm^2^ cell culture flasks were irradiated with an irradiator (XRAD320, 6 Gy) 4 h before collection. The cells were washed in PBS, and pellets were resuspended in 300 μl lysis buffer (150 mM NaCl, 25 mM Tris pH 7.5, 1 mM EDTA, 0.5% NP40, protease inhibitors (cOmplete ULTRA Tablets, Roche, Basel, Switzerland) and phosphatase inhibitors (phosphatase inhibitor cocktail 2/3, P5726/P0044, Sigma Aldrich, St Louis, MO, USA) and then incubated for 1 h at 4°C. The cell extracts were centrifuged at 13200 rpm for 20 min at 4°C. Following the measurement of the total protein amount, the supernatant fraction was incubated with 15 μl fetal bovine serum for 1 h at 4°C and then with 10 μl precleaned magnetic beads at 12 rpm for 30 min at 4°C. Next, 200 μg protein was incubated with 7.5 U of DNase I (Life Technologies, Waltham, MA, USA, #EN0521) and 1 μg anti-Flag antibody (Sigma Aldrich St Louis, MO, USA, # F3165,) at 12 rpm overnight at 4° and then incubated with precleaned magnetic beads at 12 rpm for 4 h at 4°C. The beads were washed three times with lysis buffer. Laemmli 2× (62.5 mM Tris–HCl, pH 6.8, 10% glycerol, 1% LDS and 0.005% bromophenol blue) was added, and the proteins were boiled for 5 min at 95°C. Denatured protein extracts were resolved using 4–15% Mini-PROTEAN^®^ TGX™ Precast Protein Gels (Bio-Rad, Hercules, CA, USA) and then transferred to a nitrocellulose membrane.

### Resection assay

Resection measurements on DIvA cells were performed as previously described (41). Briefly, after 4-hydroxy-tamoxifen treatment, cells were collected for genomic DNA extraction (DNeasy blood & tissue kit, Qiagen, Hilden, Germany), and 100–200 ng genomic DNA was treated with 16 U of the appropriate restriction enzyme overnight at 37°C. After enzyme inactivation, the digested genomic DNA was used for qPCR (mix for qPCR, TAKARA, Shiga, Japan) with the primers listed in the Table 1.

**Table 1.** Resection assay

DSB localization	Enzyme	Distance from DSB	FW sequence	REV sequence
chr 20	BanI	508 nt	GGGGCCATCTTCCTTTAAGA	CCAGACGCTGCCAAATAGTG
		740 nt	GTCCCCTCCCCCACTATTT	ACGCACCTGGTTTAGATTGG
		2000 nt	GTTCCTGTTATGCGGGTGTT	TGGACCCCAAATTCCTAAAG
chr 1	BamH1	364 nt	CCAGCAGTAAAGGGGAGACAGA	CTGTTCAATCGTCTGCCCTTC
		1754 nt	GAAGCCATCCTACTCTTCTCACCT	GCTGGAGATGATGAAGCCCA
		3564 nt	GCCCAGCTAAGATCTTCCTTCA	CTCCTTTGCCCTGAGAAGTGA

The percentage of ssDNA was calculated with the following equation: ssDNA % = 1/(2^(△Ct – 1) + 0.5) × 100, where △Ct = Ct of the digested sample – Ct of the nondigested sample.

### Single strand annealing assay

All reactions were performed in a buffer containing 10 mM Tris–HCl (pH 8), 50 mM sodium chloride, 2 mM calcium chloride, 1 mM DTT and 1 mM ATPγs. In reaction A, 0.1 μM (8 μM nucleotides) of a 178-mer oligonucleotide (5′-ATCATCACCATCACCATTGAGTTTAAACCCGCTGATCAGCCTCGACTGTGCCTTCTAGTTGCCAGCCATCTGTTGTTTGCGGTTCCCAACGATCAAGGCGAGTTACATGATCCCCCATGTTGTGCAAAAAAGCGGTTAGCTCCTTCGGTCCTCCGATCGTTGTCAGAAGTAAGTTGGC-3′) was preincubated with 2.6 or 8 μM WTRAD51 (ratio protein/nt: 1/3 or 1/1, respectively) for 10 min at 37°C in a final volume of 5 μl. In reaction B, 0.1 μM of a 3′-Cy5-labeled 80-mer oligonucleotide, which was complementary to the 5′-portion of the 178-mer described in reaction A (5′-GCAAACAACAGATGGCTGGCAACTAGAAGGCACAGTCGAGGCTGATCAGCGGGTTTAAACTCAATGGTGATGGTGATGAT-Cy5-3′), was preincubated under the same conditions with 2.6 or 8 μM WTRAD51 (ratio protein/nt: 1/3 or 1/1, respectively). Reactions A and B were mixed together for 5 min at 30°C. Then, 5 μM unlabeled DNA (80-mer) was added to the reaction. The total reaction was stopped by addition of 1% SDS (w/v) and 25 mM EDTA and deproteinized (30-min incubation at 37°C with 2 mg/ml proteinase K). The samples were run in a 3% (w/v) agarose gel at 80 V for 30 min in 0.5× TAE buffer. Fluorolabeled DNA species were visualized by fluorescence imaging using a Typhoon FLA 9500 (GE Healthcare Life Sciences, Marlborough, MS, USA).

### Foci counts by ImageJ

Foci were automatically counted with ImageJ using the following method.

To define a mask with nuclei in the DAPI channel, Image/Adjust/Threshold/Analyze/Analyze Particles was used. In the foci channel, all particles in the ROI manager were selected, followed by ‘OR (Combine)’. All nuclei are outlined in the foci image. To define the threshold for counting foci, we used Process/Find Maxima and ‘Single Points’ as the output type, and we determined the correct value for detecting the majority of foci (this value should not vary between images in the same experiment). A new window then appeared. In the ROI Manager, ‘Measure’ was used. In the results datasheet, the foci number by nucleus was obtained by dividing the ‘Raw Integrated Density number’ value by the ‘Max’ value (255).

### Statistical analysis

Unpaired *t*-tests were performed using GraphPad Prism 3.0 (GraphPad Software).

## RESULTS

### RAD51 and BRCA2 repress both SSA and A-EJ in an epistatic manner

**Figure 1.**
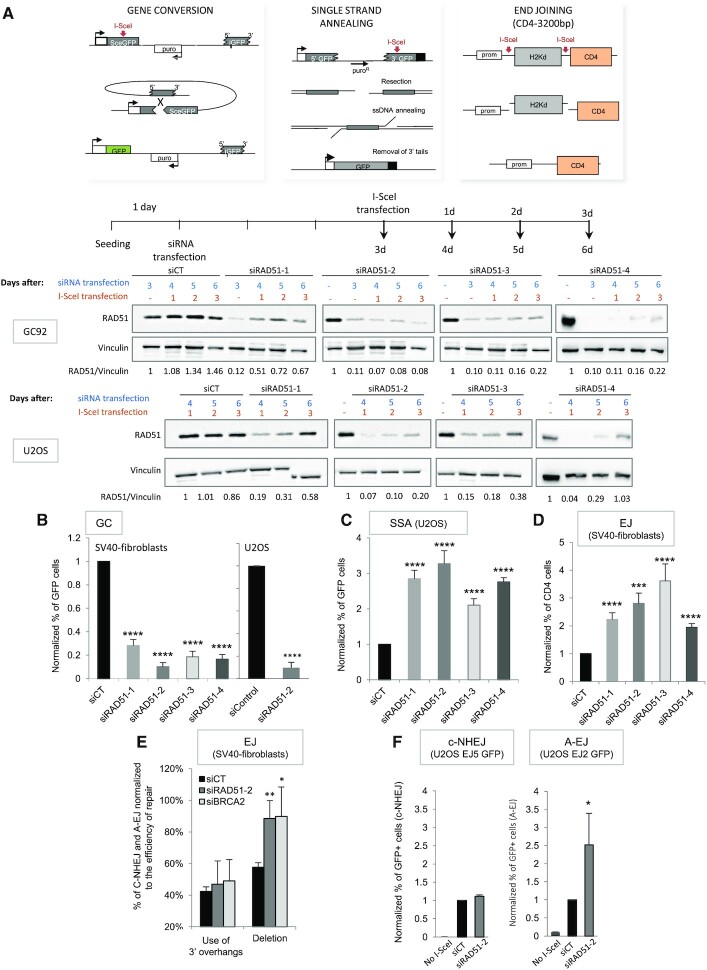
Impact of RAD51 on the GC *versus* SSA and A-EJ balance. (**A**) Upper panel: Three intrachromosomal substrates were used to monitor gene conversion (GC), single-strand annealing (SSA) or EJ (both C-NHEJ and A-EJ) on DSBs generated by meganuclease I-SceI. Lower panel: Efficiency of the four RAD51 siRNAs during the course of the experiment in SV40-transformed fibroblasts and in U2OS cells. (**B–D**). Impact of RAD51 siRNAs on gene conversion (B), SSA (C) and EJ (D). The values are shown normalized to the control siRNA (in black) and represent the average ± SEM of at least five independent experiments. (**E**) Sequences of the repair junctions from the EJ events after RAD51 or BRCA2 depletion. Use of the 3′-overhang generated by I-SceI corresponds to C-NHEJ, while A-EJ generates deletions at the junction (5,6,13,36). (**F**) Impact of RAD51 depletion on C-NHEJ and A-EJ measured in U2OS cells containing the EJ5-GFP and EJ2-GFP reporters. (*P< 0.05, **P< 0.01, ***P < 0.001, ****P< 0.0001, *t*-test compared to ‘siCT’).

We used human cell lines containing reporter substrates to monitor GC, SSA or end-joining (EJ) (Figure 1A). First, we verified that the four different siRNAs targeting RAD51 (Figure 1A) efficiently silenced RAD51 during the course of I-SceI expression (Figure 1A and Supplementary data S1A). SiRNA-mediated depletion of RAD51 inhibited GC in two different backgrounds, SV40-transformed fibroblasts or U2OS cells (Figure 1B, Supplementary data S1B), and, as expected, stimulated SSA (Figure 1C). In addition, RAD51 silencing also stimulated total EJ (Figures 1D, Supplementary data S1B). To discriminate between C-NHEJ and A-EJ, we sequenced the repair junctions in the CD4-3200bp EJ reporter substrate. Indeed, in cell lines deficient for the key C-NHEJ actors KU and XRCC4, we have demonstrated that deletions at the repair junctions are a signature of A-EJ, while the use of 3′-overhangs generated by I-SceI cleavage is a signature of C-NHEJ (Supplementary data S2) (5,6,13,36,42). RAD51 silencing increased the frequency of deletions at EJ junctions, while it had no impact on the frequency of use of 3′-overhangs (Figure 1E and Supplementary data S3), showing that RAD51 preferentially suppressed A-EJ rather than C-NHEJ. To confirm these conclusions, we used two other reporters specific of C-NHEJ (EJ5-GFP) or A-EJ (EJ2-GFP) in U2OS cells (38). RAD51 silencing did not affect C-NHEJ efficiency, while it stimulated A-EJ (Figure 1F), confirming the data obtained with the CD4-3200bp substrate in SV40-transformed human fibroblasts (Figure 1E).

**Figure 2.**
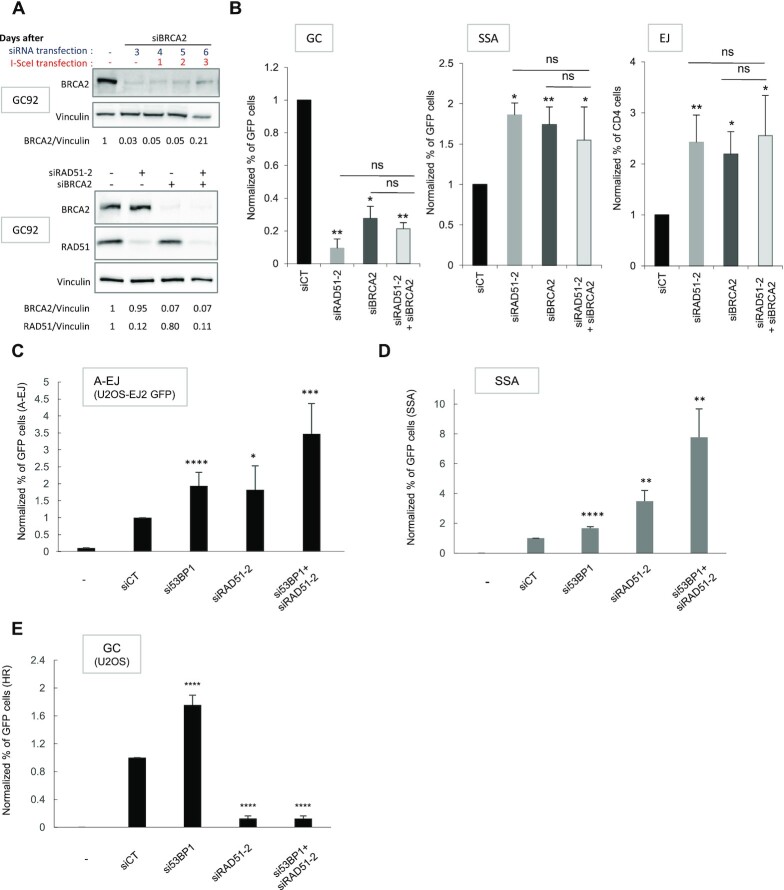
Impact of codepletion of RAD51 with BRCA2 or 53BP1 on GC, SSA and EJ. (**A**) Silencing of RAD51 and BRCA2. (**B**) Impact of simultaneous depletion of RAD51 and BRCA2 on gene conversion (GC), SSA, and EJ. The values are shown normalized to the control siRNA (in black) and represent the average ± SEM of at least 5 independent experiments. (C–E) Impact of simultaneous depletion of RAD51 and 53BP1 on A-EJ measured with the EJ2 reporter (**C**), SSA (**D**) and gene conversion (**E**). The values are shown normalized to the control siRNA (in black) and represent the average ± SEM of at least three independent experiments. (*P< 0.05, **P< 0.01, ***P < 0.001, ****P< 0.0001, *t*-test compared to ‘siCT’).

One still unresolved question is whether, in human cells, RAD51 and BRCA2 actually act in an epistatic manner not only to promote GC but also to repress SSA and A-EJ. Here, in human cells, BRCA2 depletion (Figure 2A) inhibited GC and stimulated both SSA and EJ (Figure 2B). Silencing both BRCA2 and RAD51 (Figure 2A) showed that they acted epistatically not only to promote GC (Figure 2B) but also to repress both SSA and EJ (Figure 2B). Moreover, by sequencing EJ junctions, we found that in human cells, BRCA2 depletion specifically stimulated deletions, i.e. mainly A-EJ, rather than C-NHEJ (Figure 1E and Supplementary data S2 and S3). Together, these data showed that RAD51 and BRCA2 acted in an epistatic manner not only to promote GC, as expected, but also to repress the nonconservative SSA and A-EJ.

Collectively, these data showed that RAD51 triggered conservative GC and repressed nonconservative SSA and A-EJ, all of which requiring resection of the DSB at early steps. The first step of selection of the DSB repair process involves competition between C-NHEJ and resection (4,7,13). 53BP1 inhibits resection and thereby fosters C-NHEJ. Using the CD4-3200 bp substrate, we showed that silencing 53BP1 stimulated A-EJ (43). We confirmed this result in U2OS cells using the EJ2 reporter (Figure 2C). In agreement with its role in preventing resection initiation, 53BP1 depletion also stimulated both SSA and GC (Figure 2D and E). Notably, co-depletion of RAD51 with 53BP1 further stimulated the occurrence of SSA and A-EJ (Figure 2C, D), suggesting that 53BP1 and RAD51 acted at different steps to repress SSA and A-EJ.

### Stimulation of SSA and A-EJ is separable from GC inhibition

**Figure 3.**
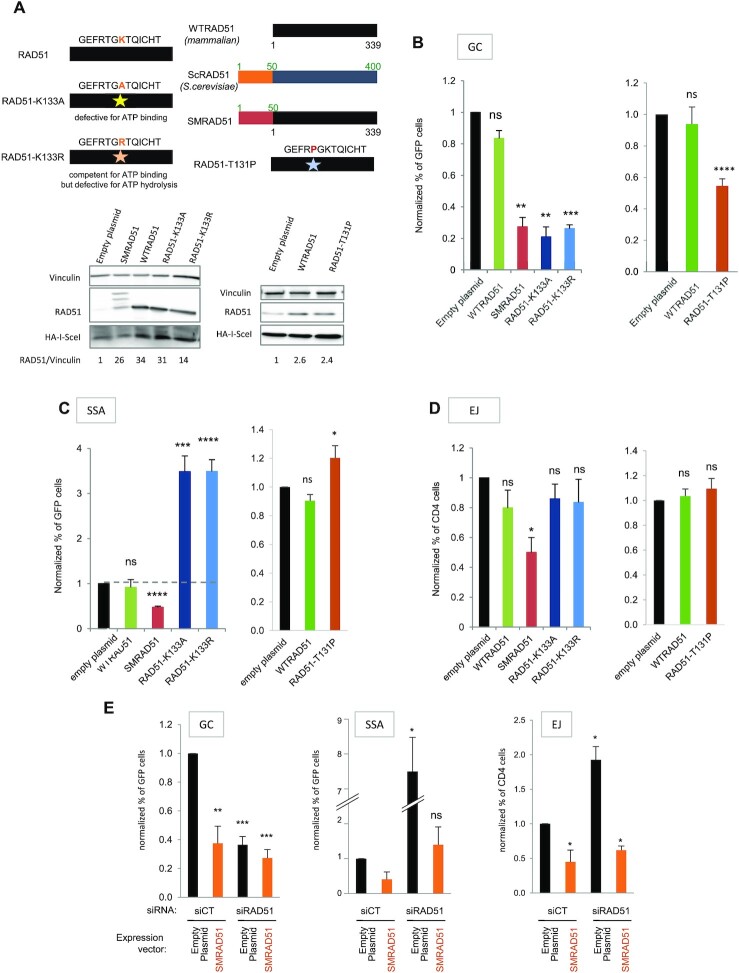
Impact of the different RAD51 dominant negative forms on DSB repair. (**A**) The different RAD51 dominant negative forms: ATPm-RAD51s mutated on the K133 ATP binding site, SMRAD51 chimera; WTRAD51 (mammalian), *Saccharomyces cerevisiae* ScRAD51 and the chimera SMRAD51 are aligned, revealing a block of 50 N-ter amino acids (AAs) present in scRAD51 but absent from WTRAD51. SMRAD51 corresponds to the fusion of 50 N-ter aminoacids from yeast to full-length WTRAD51 (11). RAD51-T131P corresponds to a mutation described in a patient suffering from Fanconi anemia group R (49). Lower panel: Expression of the different RAD51 dominant-negative forms. (B–D) Impact of the different RAD51 dominant-negative forms on GC (**B**), SSA (**C**) and EJ (**D**). The values are shown normalized to the control transfected with an empty vector (in black) and represent the average ± SEM of at least 5 independent experiments. (**E**) Impact of mixed endogenous RAD51/SMRAD51 on gene conversion, SSA and EJ. SMRAD51 was expressed in cells with silenced endogenous RAD51. The values are shown normalized to the control siRNA (in black) and represent the average ± SEM of at least 5 independent experiments. (*P< 0.05, **P< 0.01, ***P < 0.001, ****P< 0.0001, *t*-test compared to ‘empty plasmid’).

To strengthen the above data and to investigate whether RAD51 inhibited SSA and A-EJ only by channeling DSB repair toward GC or through another putative mechanism, we expressed four different DN-RAD51s that exhibited different characteristics (Figure 3A). Two of them are mammalian RAD51 mutated in the ATP binding/hydrolysis site (ATPm-RAD51s); one mutant (RAD51-K133A) does not bind ATP, and the other (RAD51-K133R) binds but does not hydrolyze ATP (24). It was shown *in vitro* that RAD51-K133A binds to DNA but fails to form filaments when RAD51-K133R forms organized filaments but is defective for RAD51 turnover (44). The third DN-RAD51 (SMRAD51 from *Saccharomyces*-mammalian RAD51) corresponds to a yeast/mammalian RAD51 chimera that harms GC (11,45–48). More recently, another DN-RAD51, RAD51-T131P, was described in a patient suffering from Fanconi anemia group R, thus corresponding to an existing pathological situation (49).

Remarkably, although all DN-RAD51s inhibited GC (Figure 3B), as expected (11,24,49), they impacted SSA and EJ differently (Figure 3C and D). Similar to RAD51 siRNA, both ATPm-RAD51s increased SSA (compare Figure 1C and Figure 3C). Overexpression of RAD51-T131P also significantly stimulated SSA (Figure 3C), although with a lower efficiency. ATPm-RAD51s and RAD51-T131P had no impact on the EJ efficiency (Figure 3D). In contrast to siRNAs and ATPm-RAD51s or RAD51-T131P, SMRAD51, stimulated neither SSA nor EJ and even slightly decreased them (Figure 3C and D), although it repressed GC (Figure 3B). These last data with SMRAD51 revealed that inhibition of GC did not systematically stimulate the alternative pathways SSA and A-EJ. Moreover, the stimulation of SSA and A-EJ resulting from silencing of endogenous RAD51 (siRNA targeting the 3′-UTR of RAD51) was abolished by ectopic expression of SMRAD51, while it failed to restore GC (Figure 3E). This finding provides further evidence of a separation of functions between GC promotion and RAD51-mediated protection against nonconservative SSA and A-EJ.

While EJ is active throughout the cell cycle (50), HR and resection are regulated by the cell cycle (41,51). Therefore, we verified the impact of the transfection of siRNAs or of the DN-RAD51s plus I-SceI-encoding plasmid on the cell cycle distribution in the two cell types we have used, U2OS and SV40-immortalized fibroblasts (Supplementary data S4). When specifically examining the proportion of cells in the S and G2/M phases, during which resection and HR are more efficient, we found that RAD51 or BRCA2 depletion altered the cell cycle distribution, leading to a slight accumulation of cells in S/G2/M (Supplementary data S4A). In contrast, the expression of DN-RAD51s did not substantially affect the cell cycle distribution (Supplementary data S4B). As ATPm-RAD51s strongly stimulate SSA (and RAD51-T131P to a lesser extent) without inducing cell cycle accumulation in resection-prone cell cycle phases, we concluded that cell cycle alteration could not account for the observed increase in SSA.

One hypothesis to account for the differences between the different DN-RAD51s proposes that although the ATPm-RAD51 proteins have been shown to bind DNA *in vitro* (44,52), SMRAD51, ATPm-RAD51s and RAD51-T131P may differ in their capacities to bind damaged DNA in living cells.

### The loading of RAD51 on damaged chromatin requires ATP in living cells

To address this hypothesis, we compared the capacities of DN-RAD51s to bind damaged DNA *in vivo*. As anti-RAD51 antibodies revealed both endo- and exogenous RAD51, we constructed Flag-RAD51s to specifically distinguish the exogenous forms.

**Figure 4.**
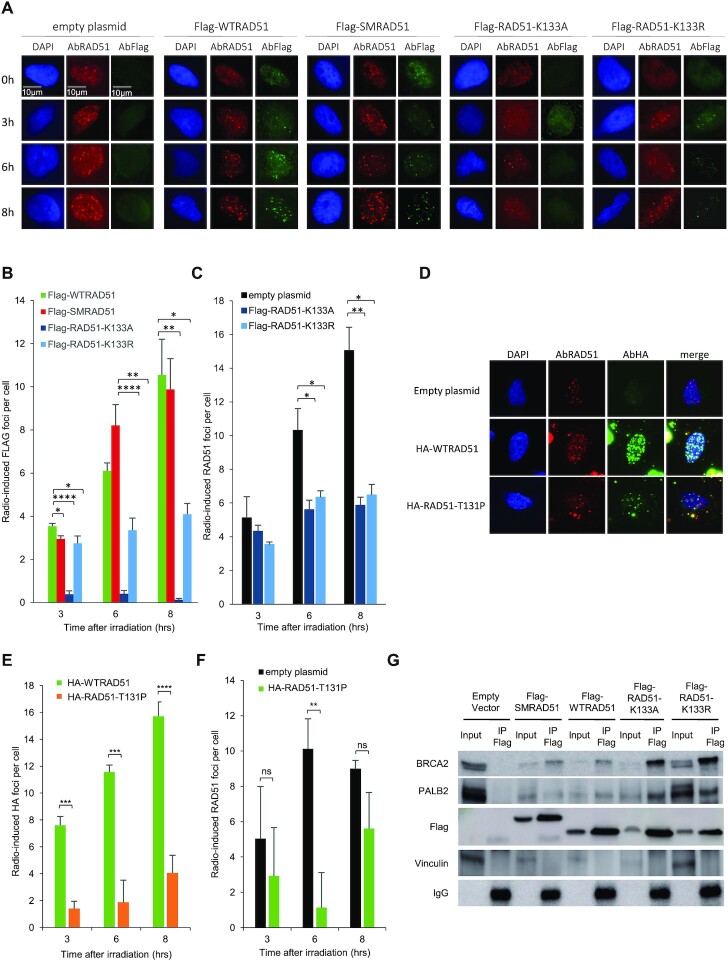
Binding of the different RAD51 dominant-negative forms on damaged DNA. (**A**) Examples of RAD51 foci at different times after IR (6 Gy) revealed by anti-Flag or anti-RAD51 antibodies. (**B**, **C**) Kinetics of IR-induced RAD51 foci upon WTRAD51, SMRAD51 or ATPm-RAD51 overexpression, revealed with an anti-Flag antibody (exogenous RAD51, panel B) or an anti-RAD51 antibody (endogenous + exogenous RAD51, panel **C**). The values represent the average ± SEM of at least three independent experiments. (D–F) Kinetics of IR-induced RAD51 foci (representative pictures, panel **D**) on RAD51-T131P overexpression revealed with an anti-HA antibody (exogenous RAD51, panel **E**) or an anti-RAD51 antibody (endogenous + exogenous RAD51, panel **F**). The values represent the average ± SEM of at least three independent experiments. (**G**) Coimmunoprecipitation experiments. Four hours after 6 Gy irradiation, cell extracts were immunoprecipitated with a Flag antibody. BRCA2 and PALB2 were then detected by western blotting (*P< 0.05, **P< 0.01, ***P < 0.001, ****P< 0.0001, *t*-test).

We first analyzed the formation of RAD51 foci induced by ionizing radiation (IR) (Figure 4A) at 6Gy, a dose corresponding to the beginning of the plateau of RAD51 foci dose response, in our cell line (Supplementary data S5). Flag-SMRAD51 efficiently formed IR-induced foci with the same kinetics as endogenous RAD51 or overexpressed Flag-WTRAD51 (Figure 4A–C). In contrast, the two ATPm-RAD51s exhibited defects in IR-induced Flag foci formation (Figure 4A and B). Indeed, Flag-RAD51-K133A did not assemble into foci, and the efficiency of foci formation was strongly decreased for Flag-RAD51-K133R (Figure 4A and B). These data were confirmed by ChIP experiments on Asi-SI endonuclease-induced DSBs, confirming the ability of SMRAD51 to bind DSBs and the poor efficiency of ATPm-RAD51 (Supplementary data S6). Moreover, in immunofluorescence experiments, the RAD51 antibody that recognizes both endogenous and exogenous RAD51 revealed that both ATPm-RAD51s affected the efficiency of all RAD51 foci formation, thus including endogenous RAD51, accounting for their dominant-negative effect (Figure 4A and C). As expected (49), HA-RAD51-T131P was defective in radiation-induced focus formation (Figure 4D and E) but also revealed an inhibitory effect on the formation of endogenous RAD51 foci (Figure 4D and F). As ATPm-RAD51s have impaired ATPase activity and RAD51-T131P has deregulated ATPase activity (49), these data directly couple ATPase activity and the ability to form RAD51 foci *in vivo*.

As ATPm-RAD51s do not bind efficiently to broken DNA *in vivo* and because BRCA2/PALB2 loads RAD51 onto ssDNA, we hypothesized that ATP might be required for the binding of RAD51 to BRCA2/PALB2. In coimmunoprecipitation analyses, WTRAD51, SMRAD51, and ATPm-RAD51s interacted with both BRCA2 and PALB2, showing that ATP hydrolysis was not required for these interactions (Figure 4G). These findings also suggested that ATPm-RAD51s could titrate endogenous HR proteins such as BRCA2 and PALB2, providing an explanation for the inhibition of endogenous RAD51 foci formation and their dominant negative effect.

Altogether, these data show that (i) DN-RAD51s that do not bind DNA in cells stimulate SSA, similarly to siRAD51 or siBRCA2, while (ii) SMRAD51 that binds damaged DNA represses GC but does not stimulate SSA and A-EJ. These findings support the conclusion that the repression of SSA and A-EJ is correlated to the binding capacities of RAD51 to damaged DNA rather than to GC proficiency (compare Figures 3 and 4).

### PARI and FBH1 helicases decrease HR in the presence of RAD51, but act differently on SSA

Our data show that the presence or the absence of RAD51 on DNA is important at the second step of the selection of the DSB repair pathway. Based on the model of SRS2 in yeast, several helicases have been proposed to remove RAD51 from DNA; including the family of the five REC Q helicases, RTEL, FBH1, PARI. One can propose that they might counteract the protective effect of RAD51 against nonconservative SSA. To test this hypothesis, we overexpressed two different helicases, PARI and FBH1, upon overexpression of WTRAD51 (that binds damaged DNA) *versus* RAD51-K133A (that does not bind DNA and prevents the binding of endogenous RAD51). Overexpression of PARI slightly decreased HR (Supplementary data S8) and did not show any further HR inhibition in the presence of RAD51-K133A that suppresses the binding of RAD51 to damaged DNA (Supplementary data S8A). This suggests that PARI is capable of inhibiting HR by removing RAD51 from the DNA. However, overexpression of PARI had no impact on SSA. As PARI is targeted to chromatin via its interaction with PCNA (53), PARI should act when strand invasion and HR are already engaged, i.e. after the choice between HR and SSA.

Overexpression of FBH1 strongly decreased HR, as already shown (54), but, like PARI, FBH1 had no impact on SSA in the presence of RAD51. Again, FBH1 is recruited to chromatin via its interacting domains with PCNA, when strand invasion is already engaged (54). Remarkably, FBH1 also abolished SSA stimulation by RAD51-K133A (Supplementary data S8). As RAD51-K133A prevents RAD51 loading on damaged DNA (see Figure 4), these data suggest that FBH1 might have other functions in the regulation of the choice between HR and SSA. This hypothesis is supported by the fact that FBH1 is a member of the ubiquitin protein ligase complex called SCFs (SKP1-cullin-F-box), which functions in phosphorylation-dependent ubiquitination (55).

These data confirmed that inhibition of HR is not systematically correlated to SSA stimulation, and therefore, the regulation of HR and SSA are separable, in support of our above data. These data also show that helicases that are recruited by PCNA to displace RAD51 from HR intermediates are not able to stimulate SSA, but that FBH1 and PARI differently operate, notably on SSA.

### RAD51 does not protect endonuclease-induced DSBs from resection

To account for the above data, since both SSA and A-EJ require resection and annealing steps, one can propose that the binding of RAD51 on DNA protects against extended resection, as shown for arrested replication forks (31,32), and/or impairs the annealing of complementary ssDNA, as suggested by *in vitro* studies in yeast (56,57).

**Figure 5.**
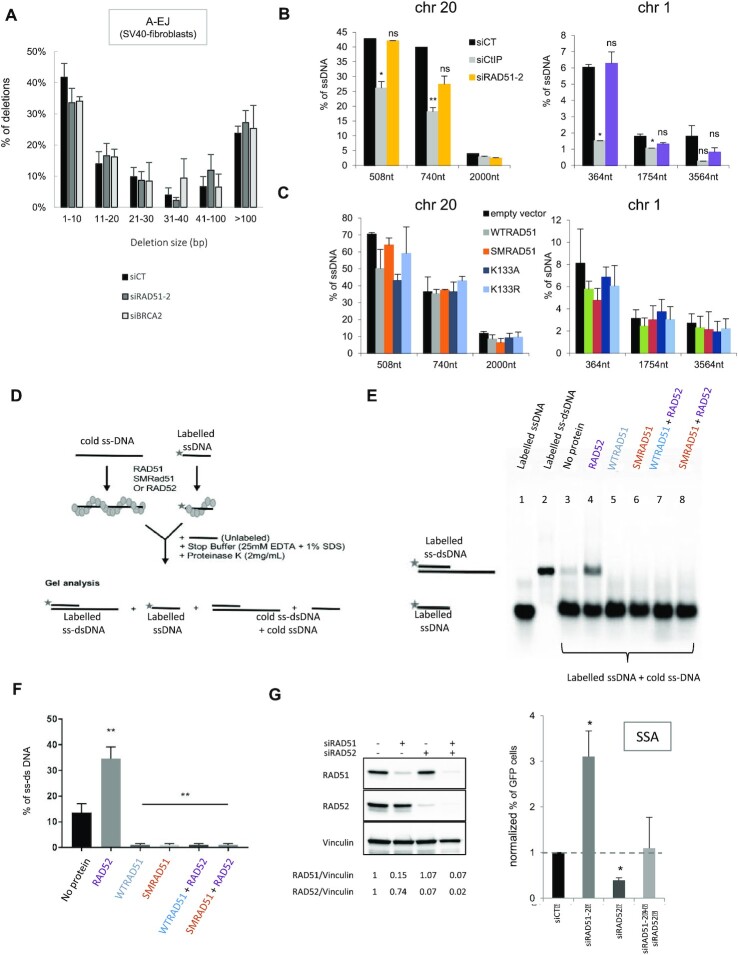
Impact of RAD51 on resection and annealing. (**A**) Impact of RAD51 or BRCA2 depletion on the size of deletions observed in A-EJ repair of the CD4-3200 bp reporter. (**B**) Impact of silencing RAD51 on resection. Two Asi-SI sites were analyzed on chromosomes 20 (58) and 1 (41). The values are the average ± SEM of at least five independent experiments. (**C**) Impact of the dominant-negative forms of RAD51 on resection. (D–F) Impact of RAD51 on the annealing of two complementary ssDNAs. (**D**) Scheme of the experiments. (**E**) Representative gel. Lanes 1 and 2 correspond to markers (ssDNA or dsDNA). Lane 3: Incubation of two naked complementary ssDNAs. Spontaneous annealing results in a band migrating at the position of the dsDNA. Lane 4: Incubation with RAD52 favors the annealing of complementary ssDNA. Lanes 5 and 6: incubation with WTRAD51 (lane 5) or SMRAD51 (lane 6) inhibits spontaneous annealing. Lanes 7 and 8: Incubation with WTRAD51 (lane 7) or SMRAD51 (lane 8) inhibits RAD52-mediated annealing. (**F**) Quantification of three experiments (histograms represent the average ± SEM). (**G**) Impact of RAD52 and RAD51 depletion (left panel) on SSA. The values (right panel) are shown normalized to the control siRNA (in black) and represent the average ± SEM of at least three independent experiments. (*P< 0.05, **P< 0.01, ***P < 0.001, ****P< 0.0001, *t*-test).

We first addressed the resection hypothesis by measuring the sizes of deletions at A-EJ junctions with the CD4-3200 bp reporter (5,6,13,36,42,43). Although silencing RAD51 and BRCA2 stimulated A-EJ (Figure 1E and F), this did not impact the distribution of the sizes of deletions (Figure 5ASupplementary data S3). Then the slight accumulation of cells in S/G2/M induced by siRAD51s and siBRCA2 was not associated with the stimulation of resection in A-EJ.

Then, we used the DIvA system, which allows to quantify the resection at different distances of Asi-SI cutting sites in the genome (41,58). We measured resection at two different sites located on chromosomes 1 and 20 that have been mapped and used in previous studies (41,58). The site on chromosome 20 was shown to be bound by RAD51 after induction of DNA double strand breaks by Asi-SI (57). Resection decreased with the distance from the Asi-S1-cleaved sites, validating this approach. In addition, as a control, silencing CtIP, which is involved in resection initiation, significantly affected the resection efficiency (Figure 5B). Neither silencing RAD51 nor expression of WTRAD51 or DN-RAD51s significantly impacted the efficiency of resection at either site (Figure 5B and C).

Since resection (which is required for RAD51 foci assembly) and HR are cell cycle dependent (41,51), we analyzed the impact of the cell cycle phases on irradiation-induced RAD51 foci formation when expressing WTRAD51 and SMRAD51 (the two RAD51 forms able to bind damaged DNA) (Supplementary data S7). The efficiencies of radiation-induced foci were similar between endogenous RAD51 and exogenous WTRAD51 or SMRAD51, when cells were irradiated in different cell cycle phases. The level of foci formation was low when the cells were irradiated in G1, in contrast to the efficient RAD51 foci formation when the cells were irradiated in S and G2, the two cell cycle phases were resection and HR are more efficient (Supplementary data S7).

Collectively, the data show that neither impairing the binding of RAD51 to DNA (with siRAD51, siBRCA2, or with ATPm-RAD51) nor expressing SMRAD51 that binds DNA but is not capable to promote GC, affect resection efficiency. Of note, these results are coherent with (i) the finding that silencing RAD51 or BRCA2 did not affect the size distribution of shorter deletions measured at the A-EJ repair junctions on the CD4-3200 bp reporter (Figure 5A); (ii) the fact that RAD51 and 53BP1 (which inhibits resection) do not act epistatically for SSA and A-EJ repression (Figure 2C and D).

Taken together, these data showed that in contrast to blocked replication forks, RAD51 did not significantly protect against long resections at DSBs generated by endonucleases.

### RAD51 prevents the annealing of complementary ssDNAs

Then, we investigated whether human WTRAD51 and SMRAD51 that both bind damaged DNA in living cells retained the ability to inhibit the annealing of complementary ssDNA *in vitro*. Two complementary ssDNAs (only one being radioactively labeled) were incubated *in vitro* with proteins, and after deproteinization, the products were analyzed by agarose gel electrophoresis (Figure 5D). In the absence of protein, the two complementary ssDNAs spontaneously annealed albeit with a low efficiency (Figure 5E and F). Incubation with human RAD52 protein stimulated the efficiency of annealing (Figure 5E and F), as expected (59), validating our assay. Both WTRAD51 and SMRAD51 abolished spontaneous and RAD52-induced annealing (Figure 5E and F). Coherently, silencing RAD52 abolished the stimulation of SSA resulting from RAD51 depletion in living human cells (Figure 5G), confirming the antagonistic roles of RAD51 and RAD52 in SSA.

## DISCUSSION

Our data reveal two separable roles for RAD51 in the choice of the DSB repair pathway (at the second step), both favoring genome stability maintenance, in human cells. In addition to triggering GC, RAD51 also prevents nonconservative SSA and A-EJ via inhibition of the annealing of complementary ssDNA. Of note, putative alterations of the cell cycle distribution cannot account for this conclusion. The increase in A-EJ and SSA upon HR deficiency has been separately reported in different studies (23,25–29), consistently proposing that DSB repair was redirected to alternative mechanisms due to the lack of strand exchange activity. Here, we show that the stimulation of SSA and A-EJ in the absence of RAD51 is not a direct consequence of the inhibition of GC *per se* but rather results from the absence of RAD51 protein on the DNA, thereby failing to repress the annealing of complementary ssDNA. PolQ has been shown to stimulate A-EJ and to inhibit the recruitment of RAD51 to ssDNA (60). This finding is highly consistent with our data and with the inhibition of A-EJ by RAD51 bound to DNA. In budding yeast, Rad51 suppresses Rad52-dependent ssDNA annealing, facilitating DNA strand invasion *in vitro* (56,57). Here, we show that, in human cells, promotion of GC and repression of annealing are in fact two separable processes of RAD51.

SSA has been described and characterized between two direct repeats flanking the DSB. One can object that such organization might be infrequent. However, SSA can occur in other situations. For example, SSA can implicate repeats as far apart as 50 kb in budding yeast (10). Therefore, direct repeats do not need to be in the immediate vicinity of the break, and limiting the length of resection is in fact a way of restricting homologous repair to gene conversion (30). SSA can also occur between inverted repeats either in intramolecular or intermolecular events (61), which is even more destabilizing for the genome. Finally, in mammalian cells, SSA between two homologous sequences located on two different chromosomes has been shown to generate translocations (19). Considering the high frequency of repeat sequences in the mammalian genome, this phenomenon might have a great impact on its stability. Indeed, in human genomes, 25% to 50% of the genome is represented by repeated sequences (depending on the publication), including LINEs and Alu sequences. The implications of these repeated elements in genome rearrangements are now clear (62), not only for intrachromosomal rearrangements but also for interchromosomal rearrangements (63). Not all repeated sequences are identical; interestingly, one study showed that RAD52 is important for rearrangements between identical Alu elements but demonstrated a reduced influence on rearrangements mediated by divergent repeats (64). Since SSA is dependent on RAD52, this finding supports the notion that such homology-dependent rearrangements arise through SSA. In addition to the stimulation of SSA, RAD51 protein ablation also fosters A-EJ, which is also necessarily mutagenic and has been implicated in translocations and numerous genomic rearrangements that are hallmarks of cancer cells (4,5,18,65–68). Therefore, tumors mutated for BRCA1/2 or RAD51 paralogs, which are deficient in the loading/stabilization of RAD51 protein on resected DNA, are prone to sustained SSA/A-EJ-mediated genomic instability.

The mutations of RAD51 that have been identified thus far in cancers are mostly coupled to altered DNA binding capacities (69,70). With regard to our findings, these mutations should foster alternative nonconservative repair pathways accounting, at least in part, for the genetic instability observed in those cells. Note that thus far, no mutation of RAD51 has been identified that impairs strand exchange activity and retains DNA binding capacities, which suggests that the activation of alternative pathways should generate genomic instability but might also be necessary for cell survival. This model is supported by the synthetic lethality that has been described between BRCA2 and Pol Theta or RAD52 (60,71).

The resection step that generates ssDNA is essential to initiate HR but is also a prerequisite for the annealing of complementary ssDNA in nonconservative SSA and A-EJ that jeopardize genome stability. The ssDNA binding protein RPA protects against annealing of microhomology–mediated A-EJ (72) but favors the annealing of long homologies catalyzed by RAD52 by preventing secondary structure formation (73,74). The function of RPA is then to direct repair to homology-dependent processes, whether GC or SSA. Our data show that ssDNA occupancy by RAD51 finalizes the selection toward GC at a later step by favoring GC through its catalytic activity and, in parallel, by inhibiting SSA through ssDNA occupancy. The regulation is made complex/subtle by the fact that helicases that are able to dismantle RAD51 binding on DNA, in HR intermediates, are unable to stimulate SSA: one can propose (i) that at the time of helicases recruitment (after strand invasion), orientation toward SSA might not be possible anymore and/or, (ii) that many helicases/translocases have been proposed as SRS-2 homologs and might have redundant functions. Overall, these processes appear to be highly and finely regulated.

*In vitro*, RAD51-K133A does not form filaments when RAD51-K133R forms proper nucleofilaments but is deficient for RAD51 turnover (44). Here, we show that in living cells, ATPm-RAD51s are inefficiently loaded onto damaged chromatin, suggesting that the primary role of ATP is the transfer of RAD51 to damaged DNA. RAD51-T131P, a dominant negative form of RAD51 found in a patient with Fanconi anemia (49), is also defective for DNA binding in living cells. This mutant has deregulated ATPase activity, confirming the coupling between ATPase activity and DNA binding in human cells.

RAD51 has been shown to protect arrested replication forks from resection (31,32). Indeed, several RAD51 mutants and separation of function alleles, including the RAD51-K133R used herein, have been shown to be able to protect arrested replication forks from resection (33,75). Since RAD51-K133R binds poorly to IR- and endonuclease-induced DSBs (Figure 4 and supplementary data S6), arrested replication forks and DSBs are distinctly processed by RAD51. This phenomenon is further supported by the resection experiments presented in Figure 5.

**Figure 6.**
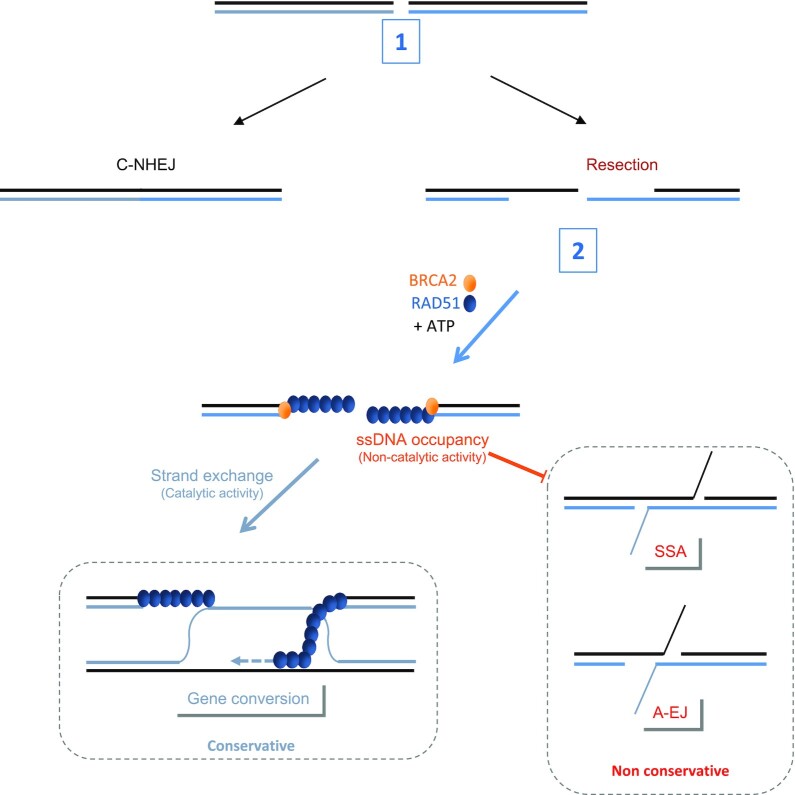
The roles of RAD51 in the selection of the DSB repair pathway. (**1**) C-NHEJ favored by 53BP1 and Ku80/70 competes with resection on DSBs. (**2**) After resection, BRCA2 loads RAD51 onto ssDNA in an ATP-dependent manner. The occupancy of ssDNA by RAD51 triggers gene conversion (in a catalytic manner) and suppresses the annealing step (independently of GC promotion), which could be RAD52-dependent, of nonconservative SSA and A-EJ. The blockage of annealing by RAD51 does not require strand exchange activity.

Altogether, our data reveal a role for RAD51 in the second step of DSB repair mechanism selection. This role requires the loading of RAD51 onto DNA, which is ATP-dependent *in vivo* and impairs the annealing of complementary ssDNA without requiring strand exchange enzymatic activity and GC. Therefore, RAD51 preserves genome stability by preventing nonconservative DSB repair through ATP-dependent but GC-independent roles (summarized in Figure 6).

More generally, because HR has been shown to be deregulated in aging- and/or cancer-prone pathological situations (Fanconi anemia, familial breast and ovary cancers, sporadic cancers), these data raise the question concerning the relative impact of HR defect compared to the increase in nonconservative mechanisms (SSA, A-EJ) on genetic stability and ultimately concerning the pathological repercussions. Remarkably, the impact on nonconservative DSB repair efficiency varies according to the mechanism of HR suppression.

## Supplementary Material

gkac073_Supplemental_File
