# Supplementary material for: RAD51 protects against nonconservative DNA double-strand break repair through a nonenzymatic function
Source: Nucleic Acids Res. 2022 Feb 8;50(5):2651–66. doi: 10.1093/nar/gkac073 (PMC8934640; doi:10.1093/nar/gkac073)
Supplement: gkac073_Supplemental_File [file gkac073_supplemental_file.docx]

**RAD51 protects against non-conservative DNA double-strand break repair, through non-enzymatic function**

Ayeong So^1,2^, Elodie Dardillac^1,2^, Ali Muhammad^3^, Catherine Chailleux^4^, Laura Sesma Sanz^5,6^, Sandrine Ragu^1,2^, Eric Le Cam^3^, Yvan Canitrot^4^, Jean Yves Masson^5,6^, Pauline Dupaigne^3^, Bernard S. Lopez^1,2^* and Josée Guirouilh-Barbat^1,2^*.

**Supplementary data**

**Supplementary data S1**

**S1A. Kinetic of I-SceI expression**

Seeding

siRNA

transfection

1 day

I-SceI

transfection

1d

2d

3d

3d

4d

5d

6d


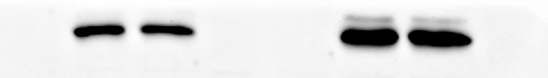

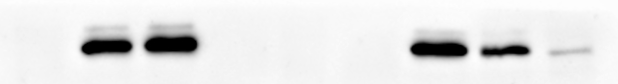


IB HA (I-SceI)


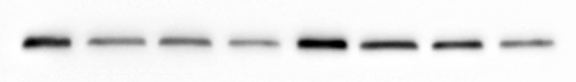


IB Tubulin


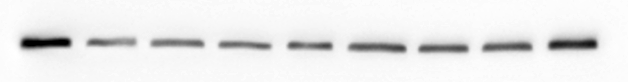


3

4

5

6

3

4

5

6

-

1

2

3

-

1

2

3

6

5

4

3

3

2

1

-

-

3

4

5

6

-

1

2

3

-

Days after :

siRNA transfection

I-SceI transfection

siRAD51-2

siCT

siRAD51-1

siRAD51-3

**Supplementary Figure S1A.** Expression of I-SceI (HA) after siRNA transfection. Upper panel: scheme of the experiment. Lower panel : The kinetic of I-SceI expression is not altered by siRNA transfection, and is maximal at 1 and 2 days after I-SceI plasmid transfection but is over at 3 days after transfection, at the time of sample collection for the FACS analysis of repair events

**S1B. Raw values of GC, SSA and EJ upon RAD51 depletion by siRNA (supplementary to figure 1B-D).**

|  | GC (%GFP cells) | | SSA (% GFP cells) | | EJ (% CD4 cells) | |
| --- | --- | --- | --- | --- | --- | --- |
|  | mean | sem | mean | sem | mean | sem |
| no I-SceI | 0.17 | 0.03 | 0 | 0 | 0.1 | 0.04 |
| **siCT** | **3.3** | **0.38** | **1.11** | **0.22** | **1.24** | **0.2** |
| siRAD51-1 | 0.77 | 0.23 | 2.65 | 0.31 | 2.22 | 0.21 |
| siRAD51-2 | 0.27 | 0.12 | 8.09 | 0.9 | 2.77 | 0.41 |
| siRAD51-3 | 0.67 | 0.18 | 3.1 | 0.66 | 6.55 | 3.5 |
| siRAD51-4 | 0.43 | 0.03 | 1.67 | 0.23 | 2.49 | 0.69 |

**Supplementary data S2**: Summary of the characterization of C-NHEJ versus A-EJ using the CD4-3200bp end joining reporter in previous studies


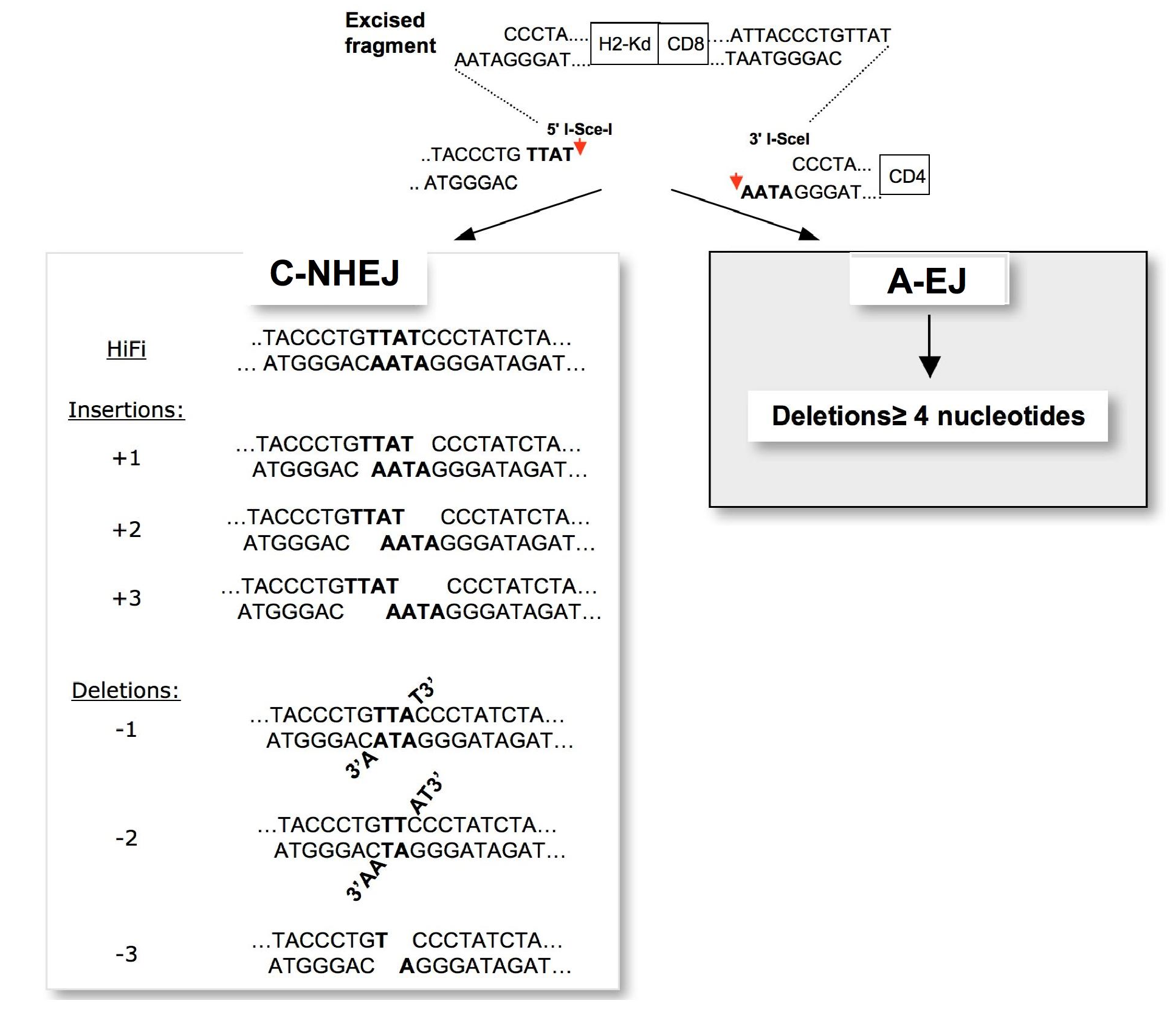


**Supplementary Figure S2: Summary of the characterization of C-NHEJ versus A-EJ using the CD4-3200bp end joining reporter in previous studies** (Guirouilh-Barbat et al., 2007; Guirouilh-Barbat et al., 2004; Rass et al., 2009). Examples of end-joining intermediates in C-NHEJ (left panel) and A-EJ (right panel). Upper panel: the structure of the I-SceI cleavage site (bold type indicates the four 3’-overhangs generated by I-SceI cleavage).

C-NHEJ and A-EJ differ in the junction patterns that occur after the resealing of the DNA ends. Whereas C-NHEJ uses the four 3’-overhangs generated by I-SceI cleavage, A-EJ is characterized by deletions at the junctions of 3’ protrusions of at least four, but usually more, nucleotides.

C-NHEJ uses the 3’-overhangs; it is able to join both fully and non-fully cohesive ends and can generate imperfect annealing. These intermediates are then processed for gap filling and mismatch repair, leading either to error-free end-joining (HiFi, perfect annealing) or the deletion or insertion of 1 to 3 nucleotides at the cleavage sites according to the intermediates shown in Supplementary Fig. S2. Such events have not been observed in *KU*- or *XRCC4*-deficient cells.

In *KU-* or *XRCC4*-deficient cells (which therefore use exclusively A-EJ), the use of the 3’-overhangs disappears, HiFi events are extremely rare, and 1- to 3-nt insertions or deletions are never observed; in contrast, the deletion of at least the 3’-overhangs (and generally more extended regions) is observed.

In conclusion, the following generalities apply:

- Deletions of more that the 3’-overhangs at the junctions (generated by I-SceI) are a hallmark of A-EJ
- The use of the 3’-overhangs is not functional in A-EJ and corresponds to C-NHEJ

Finally, capture of DNA fragments is sometimes observed in both C-NHEJ and A-EJ.

**References**

Guirouilh-Barbat, J., Rass, E., Plo, I., Bertrand, P., and Lopez, B. S. (2007). Defects in XRCC4 and KU80 differentially affect the joining of distal nonhomologous ends. Proc Natl Acad Sci U S A *104*, 20902-20907.

Guirouilh-Barbat, J., Huck, S., Bertrand, P., Pirzio, L., Desmaze, C., Sabatier, L., and Lopez, B. S. (2004). Impact of the KU80 pathway on NHEJ-induced genome rearrangements in mammalian cells. Mol Cell *14*, 611-623.

Rass, E., Grabarz, A., Plo, I., Gautier, J., Bertrand, P., and Lopez, B. S. (2009). Role of Mre11 in chromosomal nonhomologous end joining in mammalian cells. Nat Struct Mol Biol *16*, 819-824.

**Supplementary data S3**

**Supplementary data S3: Sequencing of repair scars on the CD4-3200bp reporter in GC92 cells transfected with the different siRNA.**

**siCONTROL**

Use of 3’ overhangs : 82 seq/ 202 total = 40%

CACGGAAGGAATTACCCTG- TATCCCTATCTAGATATGAAATCACGCCATGTA

CACGGAAGGAATTACCCTGTT—CCCTATCTAGATATGAAATCACGCCATGTA

CACGGAAGGAATTACCCTGT---CCCTATCTAGATATGAAATCACGCCATGTA

CACGGAAGGAATTACCCTGTTATCCCTATCTAGATATGAAATCACGCCATGTA

CACGGAAGGAATTACCCTGTTATCCCTATCTAGATATGAAATCACGCCATGTA

CACGGAAGGAATTACCCTG—ATCCCTATCTAGATATGAAATCACGCCATGTA

CACGGAAGGAATTACCCTG-TATCCCTATCTAGATATGAAATCACGCCATGTA

CACGGAAGGAATTACCCTGTTATCCCTATCTAGATATGAAATCACGCCATGTA

CACGGAAGGAATTACCCTGTTATCCCTATCTAGATATGAAATCACGCCATGTA

CACGGAAGGAATTACCCTGTTATCCCTATCTAGATATGAAATCACGCCATGTA

CACGGAAGGAATTACCCTGTTATCCCTATCTAGATATGAAATCACGCCATGTA

CACGGAAGGAATTACCCTGTTATCCCTATCTAGATATGAAATCACGCCATGTA

CACGGAAGGAATTACCCTGTTATCCCTATCTAGATATGAAATCACGCCATGTA

CACGGAAGGAATTACCCTGTTATCCCTATCTAGATATGAAATCACGCCATGTA

CACGGAAGGAATTACCCTG----CCCTATCTAGATATGAAATCACGCCATGTA

CACGGAAGGAATTACCCTGTT--CCCTATCTAGATATGAAATCACGCCATGTA

CACGGAAGGAATTACCCTGTTATCCCTATCTAGATATGAAATCACGCCATGTA

CACGGAAGGAATTACCCTGTTATCCCTATCTAGATATGAAATCACGCCATGTA

CACGGAAGGAATTACCCTG-TATCCCTATCTAGATATGAAATCACGCCATGTA

CACGGAAGGAATTACCCTGTT--CCCTATCTAGATATGAAATCACGCCATGTA

CACGGAAGGAATTACCCTG-TATCCCTATCTAGATATGAAATCACGCCATGTA

CACGGAAGGAATTACCCTGTTATCCCTATCTAGATATGAAATCACGCCATGTA

CACGGAAGGAATTACCCTGTTATCCCTATCTAGATATGAAATCACGCCATGTA

CACGGAAGGAATTACCCTGTTATCCCTATCTAGATATGAAATCACGCCATGTA

CACGGAAGGAATTACCCTGTTATCCCTATCTAGATATGAAATCACGCCATGTA

CACGGAAGGAATTACCCTGTTATCCCTATCTAGATATGAAATCACGCCATGTA

CACGGAAGGAATTACCCTGTTATCCCTATCTAGATATGAAATCACGCCATGTA

CACGGAAGGAATTACCCTGTTATCCCTATCTAGATATGAAATCACGCCATGTA

CACGGAAGGAATTACCCTGTTATCCCTATCTAGATATGAAATCACGCCATGTA

CACGGAAGGAATTACCCTGTTATCCCTATCTAGATATGAAATCACGCCATGTA

CACGGAAGGAATTACCCTG-TATCCCTATCTAGATATGAAATCACGCCATGTA

CACGGAAGGAATTACCCTGT---CCCTATCTAGATATGAAATCACGCCATGTA

CACGGAAGGAATTACCCTG-TATCCCTATCTAGATATGAAATCACGCCATGTA

CACGGAAGGAATTACCCTGTT---CCCTATCTAGATATGAAATCACGCCATGTA

CACGGAAGGAATTACCCTGTTATCCCTATCTAGATATGAAATCACGCCATGTA

CACGGAAGGAATTACCCTGTTATCCCTATCTAGATATGAAATCACGCCATGTA

CACGGAAGGAATTACCCTGTTATCCCTATCTAGATATGAAATCACGCCATGTA

CACGGAAGGAATTACCCTGTTATCCCTATCTAGATATGAAATCACGCCATGTA

CACGGAAGGAATTACCCTGTTATCCCTATCTAGATATGAAATCACGCCATGTA

CACGGAAGGAATTACCCTGTTATCCCTATCTAGATATGAAATCACGCCATGTA

CACGGAAGGAATTACCCTGTTATCCCTATCTAGATATGAAATCACGCCATGTA

CACGGAAGGAATTACCCTGTTATCCCTATCTAGATATGAAATCACGCCATGTA

CACGGAAGGAATTACCCTGTTATCCCTATCTAGATATGAAATCACGCCATGTA

CACGGAAGGAATTACCCTGTTATCCCTATCTAGATATGAAATCACGCCATGTA

CACGGAAGGAATTACCCTGTTATCCCTATCTAGATATGAAATCACGCCATGTA

CACGGAAGGAATTACCCTGTTATCCCTATCTAGATATGAAATCACGCCATGTA

CACGGAAGGAATTACCCTGTTATCCCTATCTAGATATGAAATCACGCCATGTA

CACGGAAGGAATTACCCTGTTATCCCTATCTAGATATGAAATCACGCCATGTA

CACGGAAGGAATTACCCTGTT—CCCTATCTAGATATGAAATCACGCCATGTA

CACGGAAGGAATTACCCTGTTATCCCTATCTAGATATGAAATCACGCCATGTA

CACGGAAGGAATTACCCTGTT-TCCCTATCTAGATATGAAATCACGCCATGTA

CACGGAAGGAATTACCCTGTTATCCCTATCTAGATATGAAATCACGCCATGTA

CACGGAAGGAATTACCCTGTTATCCCTATCTAGATATGAAATCACGCCATGTA

CACGGAAGGAATTACCCTG-TATCCCTATCTAGATATGAAATCACGCCATGTA

CACGGAAGGAATTACCCTGTTATCCCTATCTAGATATGAAATCACGCCATGTA

CACGGAAGGAATTACCCTGTTATCCCTATCTAGATATGAAATCACGCCATGTA

CACGGAAGGAATTACCCTGTTATCCCTATCTAGATATGAAATCACGCCATGTA

CACGGAAGGAATTCCCCTGTTATCCCTATCTAGATATGAAATCACGCCATGTA

CACGGAAGGAATTACCCTGT---CCCTATCTAGATATGAAATCACGCCATGTA

CACGGAAGGAATTACCCTGTTATCCCTATCTAGATATGAAATCACGCCATGTA

CACGGAAGGAATTACCCTGTTATCCCTATCTAGATATGAAATCACGCCATGTA

CACGGAAGGAATTACCCTGTTATCCCTATCTAGATATGAAATCACGCCATGTA

CACGGAAGGAATTACCCTGTTATCCCTATCTAGATATGAAATCACGCCATGTA

CACGGAAGGAATTACCCTG—ATCCCTATCTAGATATGAAATCACGCCATGTA

CACGGAAGGAATTACCCTGTTATCCCTATCTAGATATGAAATCACGCCATGTA

CACGGAAGGAATTACCCTGTTATCCCTATCTAGATATGAAATCACGCCATGTA

CACGGAAGGAATTACCCTGTTATCCCTATCTAGATATGAAATCACGCCATGTA

CACGGAAGGAATTACCCTGTT—CCCTATCTAGATATGAAATCACGCCATGTA

CACGGAAGGAATTACCCTGTTATCCCTATCTAGATATGAAATCACGCCATGTA

CACGGAAGGAATTCCCCTGTTATCCCTATCTAGATATGAAATCACGCCATGTA

CACGGAAGGAATTCCCCTGTTATCCCTATCTAGATATGAAATCACGCCATGTA

CACGGAAGGAATTACCCTGTTATCCCTATCTAGATATGAAATCACGCCATGTA

CACGGAAGGAATTACCCTGTTATCCCTATCTAGATATGAAATCACGCCATGTA

CACGGAAGGAATTACCCTGTTATCCCTATCTAGATATGAAATCACGCCATGTA

CACGGAAGGAATTACCCTGTTATCCCTATCTAGATATGAAATCACGCCATGTA

CACGGAAGGAATTACCCTGTTATCCCTATCTAGATATGAAATCACGCCATGTA

CACGGAAGGAATTACCCTGTTATCCCTATCTAGATATGAAATCACGCCATGTA

CACGGAAGGAATTACCCTGTTATCCCTATCTAGATATGAAATCACGCCATGTA

CACGGAAGGAATTACCCTGTTATCCCTATCTAGATATGAAATCACGCCATGTA

CACGGAAGGAATTACCCTGTTATCCCTATCTAGATATGAAATCACGCCATGTA

CACGGAAGGAATTACCCTGTT--CCCTATCTAGATATGAAATCACGCCATGTA

CACGGAAGGAATTACCCTGT---CCCTATCTAGATATGAAATCACGCCATGTA

Insertion : 2 seq/ 202 total = 1%

CACGGAAGGAATTACCCTGT-ins 107-TATCCCTATCTAGATATGAAATCACGCCATGT

CACGGAAGGAATTACCCTGT-ins T-TATCCCTATCTAGATATGAAATCACGCCATGTA

Deletion : 93 seq/ 202 total = 47%

GCTGGCTAGCGCTCTAGAGCAACA--------del 20 mhom 1-------TCCCTATCTAGATATGAAA

AGAGCAACACGGAAGGAATTACCCT-----del 9 mhom 4----ATCTAGATATGAAATCACGCCA

AGAGCAACACGGAAGGAATTACCCT-----del 9 mhom 4----ATCTAGATATGAAATCACGCCA

TAGCGCTCTAGAGCAACACG-----------del 42 mhom 4-------------------------CCATGTAGTGT

AGAGCAACACGGAAGGAATTACCCT-----del 9 mhom 4----ATCTAGATATGAAATCACGCCA

ATACGACTCACTATAGGGAGA------------del 392 mhom4-------------GTTCCCAGAAGAAG

CGTGGATAGCGGTTTGA-----------------del 289 mhom 3-----------AATCACGCCATGTAGTG

CTAGAGCAACACGGAAGGAATTACC---del 4 mhom 0-----TATCCCTATCTAGATATGAAAT

AGAGCAACACGGA---------del 13 mhom 0----TTATCCCTATCTAGATATGAAATCAC

CACGGAAGGAATTACCCTG------del 12 mhom 0------AGATATGAAATCACGCCATGTAGTG

CACGGAAGGAATTACCCT—del 2 mhom 1------TATCCCTATCTAGATATGAAATCAC

CACGGAAGGAATTACCCTG---del 5 mhom 0--CCTATCTAGATATGAAATCACGCCATG

CACGGAAGGAATTACCCT-----del 9 mhom 4----ATCTAGATATGAAATCACGCCATG

CACGGAAGGAAT-------del 11 mhom 2----CCCTATCTAGATATGAAATCACGC

CACGGAAGGAATTACCCT-----del 9 mhom 4----ATCTAGATATGAAATCACGCCATGTA

TAGCGGTTTGACTCACG----------------del 351 mhom 2----------AATTCGAGCTCGC

CACGGAAGGAATTACCCT-del 1 mhom 0-------TTATCCCTATCTAGATATGAA

CACGGAAGGAATTACCCT-----del 9 mhom 4----ATCTAGATATGAAATCACGC

CACGGAAGGAATTACCCT------del 9 mhom 4---ATCTAGATATGAAATCACGC

CACGGAAGGAAT----del 8 mhom 3 ----TATCCCTATCTAGATATGAAATC

CAACACGGAAGGAATTACCC-----------------del 191 mhom 3-----------------AACCAACAAGAGC

CACGGAAGGAATTACCCT------del 9 mhom 4---ATCTAGATATGAAATCACGCCATGT

CACGGAAGGAATTACCCT------del 9 mhom 4---ATCTAGATATGAAATCACGCCATGT

CACGGAAGGAATTACCCTGTTAT---del 4 mhom 1---ATCTAGATATGAAATCACGCCATGTA

CACGGAAGGAATTACC-----del 10 mhom 2-----TATCTAGATATGAAATCACGCCATG

GCGCTCTAGAGCAACAC-------------del 28 mhom 0-------------AGATATGAAATCACGCCATGTA

GGCTAGCGCTCTAGAGCAACACG--------------del 102 mhom 2--------AATTCGAGCTCGCCCGGG

CACGGAAGGAAT-----del 8 mhom 3---TATCCCTATCTAGATATGAAAT

CACGGAAGGAATTACCCTGT----------del 22 mhom 1------------CACGCCATGTAGTGTATTGACC

GCGCTCTAGAGCAACACGGA--------------------del 108 mhom 2-------GCTCGCCCGGGGATCCT

CACGGAAGGAATTACCCTGTTA-----del 9 mhom 0----AGATATGAAATCACGCCATGTAGTG

AGGGAGACCCAAGCTGG-----------------del 43 mhom 0---------------TATCTAGATATGAAATCAC

GCAACACGGAAGGAATTACCC-----------del 31 mhom 0---------------ATGTAGTGTATTGACCGA

TGGCTAGCGCTCTAGAGCAACA-----------del 24 mhom 0-------------TATCTAGATATGAAATCAC

CGCTCTAGAGCAACACGGAAGGAAT----del 8 mhom 3----TATCCCTATCTAGATATGAAATCACG

CTCTAGAGCAACACGGAAGG----------------del 331 mhom 6-----------GGAATCAGCAGAAGTG

GTGTACGGTGGGAGGTC---------del 138 mhom 4---------------------TAGATATGAAATCACGCCA

CACGGAAGGAATT----- del 10 mhom 1-----CCCTATCTAGATATGAAATCACGCCATGT

CACGGAAGGAATTACCCT-------del 10 mhom 1—ATCTAGATATGAAATCACGCCAT

CACGGAAGGAATTACCCT------del 9 mhom 4---ATCTAGATATGAAATCACGCCATG

CACGGAAGGAAT-------del 8 mhom 3-------TATCCCTATCTAGATATGAAA

CACGGAAGGAA----------del 28 mhom 3-------------------ATCACGCCATGTAGTGTATTG

CACGGAAGGAATTACCCT-----del 9 mhom 4----ATCTAGATATGAAATCACGCCAT

CACGGAAGGAATTACCCT—del 5 mhom 1---CCCTATCTAGATATGAAATCACGCCAT

CACGGAAGGAAT------del 8 mhom 3—TATCCCTATCTAGATATGAAATCAC

CACGGAAGGAAT----del 8 mhom 3----TATCCCTATCTAGATATGAAATCACGCCAT

CACGGAAGGAATTACCCT-----del 9 mhom 4----ATCTAGATATGAAATCACGCCATGTAG

CACGGAAGGAATTACCCT----- del 9 mhom 4----ATCTAGATATGAAATCACGCCATGTAG

CACGGAAGGAATTACCCT-------del 9 mhom 4---ATCTAGATATGAAATCACGCCATGTAGTG

CACGGAAGGAATTACCCT-------- del 9 mhom 4----ATCTAGATATGAAATCACGCCATG

CACGGAAGGAATTACCCT--------del 9 mhom 4----ATCTAGATATGAAATCACGCCATG

CACGGAAGGAATTA-------del 14 mhom 2-------TCTAGATATGAAATCACGCCATGTA

CTGGCTAGCGCTCTAGAGCA-----------del 250 mhom 0----------------CTCTCTTAGGCGCTTGC

GCTCTAGAGCAACACGG-------------del 41 mhom 1------------CCATGTAGTGTATTGACCG

ACACGGAAGGAATTACCCT------del 9 mhom 4---ATCTAGATATGAAATCAC

ACGGAAGGAATTACCCTGTTA-----del 8 mhom 1---TAGATATGAAATCACGCCATGTAGTGT

CACGGAAG-------------------del 30 mhom 1-----------AAATCACGCCATGTAGTGTA

CCATTGACGCAAATG-----------------del 315 mhom 2---------GGAAGGACTGGCCAGAGG

CACGGAAGGAAT------del 8 mhom 3—TATCCCTATCTAGATATGAAATCAC

CTAGAGCAACACGGAAGG-------------del 330 mhom 6------------------GGAATCAGC

CACGGAAGGA------del 16 mhom 0 ----------TATCTAGATATGAAATCACGCCATG

TGGGAGGTCTATATA----------del 23 mhom 2-------------AGAACCCACTGCTTACTGGCTTATCGA

CACGGAAGGAAT---------del 15 mhom 1------ATCTAGATATGAAATCACGCCATGTAGT

CACGGAAGGAAT-----------del 23 mhom 2------------ATGAAATCACGCCATGTAGTGTA

CACGGAAGGAATTACCCT----del 9 mhom 4-----ATCTAGATATGAAATCACGC

ACGGAAGGAATTACCCT------del 9 mhom 4---ATCTAGATATGAAATCACGCCATGTA

ACGGAAGGAATTACCCT------del 9 mhom 4---ATCTAGATATGAAATCACGCCATGTA

CACGGAAGGAATTACCCT----del 9 mhom 4-----ATCTAGATATGAAATCACGCCATGTAG

CACGGAAGGAATTACCCT----del 9 mhom 4-----ATCTAGATATGAAATCACGCCATGTAG

CACGGAAGGAATTACCCTGT-----del 9 mhom 1----CTAGATATGAAATCACGCCATGTA

CACGGAAGGAAT-----del 8 mhom 3---TATCCCTATCTAGATATGAAA

CACGGAAGGAATTACCCTGTTAT-----del 14 mhom 3---------GAAATCACGCCATGTAGTGTAT

ACCCAAGCTGGCTA-------------- del 42 mhom 3----------TCTAGATATGAAATCA

AATACGACTCACTATAGGGAG--------------------del 117 mhom 4-----------ACCCGGTCAC

CACGGAAGGAATTACCCT----del 9 mhom 4-----ATCTAGATATGAAATCACGCCATGTAG

GCTAGCGCTCTAGAGCAA -------del 290 mhom 3----------CAACTCCTAGCTGTCACTCAAGG

CACGGAAGGAAT---del 8 mhom 3-----TATCCCTATCTAGATATGAAATCACGCC

GCTAGCGCTCTAGAGCAACACGG-------------del 51 mhom 1--------------TATTGACCGATTCCTT

CACTATAGGGAGACCC--------------del 50 mhom 3---------------TATCTAGATATGAAATCACGCC

AGGGAGACCCAAGCTGGC----------------del 46 mhom 4------------TAGATATGAAATCACG

CTTACTGGCTTATCGAAATTA----------------------del 394 mhom 1------------GCAGAACTGCCCTG

CACGGAAGGAATTACCC—del 3 mhom 1-----TATCCCTATCTAGATATGAAATCACGCCA

GCGTGTACGGTGGGAGGTC---------del 138 mhom 4--------------TAGATATGAAATCACGC

CACGGAAGGAAT---del 8 mhom 3-----TATCCCTATCTAGATATGAAATC

CACGGAAGGA-------del 13 mhom 0------CCCTATCTAGATATGAAA

CAAGTCTCCACCCCAT------------del 247 mhom 1-----------ATCTAGATATGAAATCACGCCA

CACGGAAGGAATTACCCTGTTATC---del 6 mhom 4---TAGATATGAAATCACGCCAT

CACGGAAGGAATTACCCTGT------del 9 mhom 1---CTAGATATGAAATCACGCCATGTA

CACGGAAGGAATTACCCT------del 9 mhom 4---ATCTAGATATGAAATCACGCCAT

TATAGGGAGACCCAAGCTGGCT------------del 342 mhom 3----------GGGGAAGGAAGGGGAAT

ACGGAAGGAATTACCC------------------del 195 mhom 2-----------AACAAGAGCTCAAGGAGACC

CACGGAAGGAATT----------------del 31 mhom 0---------------CGCCATGTAGTGTATTGACCGA

GGAGACCCAAGCTGGCT-------------del 342 mhom 3-----------GGGGAAGGAAGGGGAATCAG

Deletion / Insertion : 25 seq/ 202 total = 12%

AGCGCTCTAGAGCAACACGGA-----del 316 / ins TAT--------GTGCTGGGGAAGGAAGGG

CACGGAAGGAATT----del 7 / ins CA---TATCCCTATCTAGATATGAAATCAC

AGCAACACGGAAGGAATT--------------del 276 / ins T -----------CAACTCCTAGCTGTCA

CACGGAAGG-GT-----del 15 / ins GTAATTA—CCTATCTAGATATGAAATCACGCCAT

GAGACCCAAGCTGGCTAG---------------del 36 / ins GT -----------------CCTATCTAGATATG

CACGGAAGGAATT----del 11/ins T------CCTATCTAGATATGAAATCACGCCA

AGCGCTCTAGAGCAAC----------del 366 / ins TGCCCTGCGAGAG-----------TCCCAGAAGAAGATCACA

CACGGAAGG---------del 27 / ins G------------------GAAATCACGCCATGTAGTGTATTG

AGCGCTCTAGAGCAACACG-------del 354 / ins TGTTGTT--------------CTGCGAGAGTTCCCAGA

AAGCTGGCTAGCGCTCTAGA---------------del 30 / ins TATGAA---ATCTAGATATGAAATCACGCCA

AAGGAATTACCCTGTTATCC--------------del 36 / ins TTCCTGTT –ACCGATTCCTTGCGGTCCGAATG

GGAATTACCCTGTTA----del 1 / ins GCGTTTTAACAAATTTCCGTC ----CCCTATCTAGATATGAAA

CACGGAAGGAATTA----del 16 / ins GA--------TAGATATGAAATCACGCCATGTAGTGT

CACGGAAGG------del 14 / ins TTA-------CCCTATCTAGATATGAAATCACGCCATG

CACGGAAGGA- del 20 / ins TATGA--------TAGATATGAAATCACGCCAT

TGGCTAGCGCTCCAGAGCAACA ---del 24 ins TAGGTCA-----TATCTAGATATGAAATCACGC

CACGGAAGGAATTACCCTGT---del 6 / ins A----TATCTAGATATGAAATCACGCCATGTA

GGTGATGCGGTTTTGGC-----------------del 712 / ins 154---GC

TGGTGATGCGGTTTTGGC---------del 712 / ins 154-----------GC

GGTGATGCGGTTTTGGC----------del 709 / ins 155---------- TGA

GGAAGGAATTACCCTGTTAT--------del 5 / ins 107----------TCTAGATATG

ATGCGGTTTTGGC--------------------- del 710 / ins 282-------------------GCA

GGTGATGCGGTTTTGGC------del 711 / ins 154-------------CTG

AACACGGAAGGAATT---del 26 / ins 112--------------AATCACGCCATGTAGT

CTGGCTAGCGCTCTAGAGCAACAC----del 22 / ins 33---CTATCTAGATATG

**si RAD51-2**

Use of 3’ overhangs : 57 seq/ 191 total = 30%

CACGGAAGGAATTACCCTGTTATCCCTATCTAGATATGAAATCACGCCATGTA

CACGGAAGGAATTACCCTGTTATCCCTATCTAGATATGAAATCACGCCATGTA

CACGGAAGGAATTACCCTGTTATCCCTATCTAGATATGAAATCACGCCATGTA

CACGGAAGGAATTACCCTGTTATCCCTATCTAGATATGAAATCACGCCATGTA

CACGGAAGGAATTACCCTGTTATCCCTATCTAGATATGAAATCACGCCATGTA

CACGGAAGGAATTACCCTGTTATCCCTATCTAGATATGAAATCACGCCATGTA

CACGGAAGGAATTACCCTGTTATCCCTATCTAGATATGAAATCACGCCATGTA

CACGGAAGGAATTACCCTGTTATCCCTATCTAGATATGAAATCACGCCATGTA

CACGGAAGGAATTACCCTGTTATCCCTATCTAGATATGAAATCACGCCATGTA

CACGGAAGGAATTACCCTGTTATCCCTATCTAGATATGAAATCACGCCATGTA

CACGGAAGGAATTACCCTGTTATCCCTATCTAGATATGAAATCACGCCATGTA

CACGGAAGGAATTACCCTGTTATCCCTATCTAGATATGAAATCACGCCATGTA

CACGGAAGGAATTACCCTGTTATCCCTATCTAGATATGAAATCACGCCATGTA

CACGGAAGGAATTACCCTGTTATCCCTATCTAGATATGAAATCACGCCATGTA

CACGGAAGGAATTACCCTGTTATCCCTATCTAGATATGAAATCACGCCATGTA

CACGGAAGGAATTACCCTGTTATCCCTATCTAGATATGAAATCACGCCATGTA

CACGGAAGGAATTACCCTGTTATCCCTATCTAGATATGAAATCACGCCATGTA

CACGGAAGGAATTACCCTGTTATCCCTATCTAGATATGAAATCACGCCATGTA

CACGGAAGGAATTACCCTGTTATCCCTATCTAGATATGAAATCACGCCATGTA

CACGGAAGGAATTACCCTGTTATCCCTATCTAGATATGAAATCACGCCATGTA

CACGGAAGGAATTACCCTGTTATCCCTATCTAGATATGAAATCACGCCATGTA

CACGGAAGGAATTACCCTGTTATCCCTATCTAGATATGAAATCACGCCATGTA

CACGGAAGGAATTACCCTGTTATCCCTATCTAGATATGAAATCACGCCATGTA

CACGGAAGGAATTACCCTGTTATCCCTATCTAGATATGAAATCACGCCATGTA

CACGGAAGGAATTACCCTGTTATCCCTATCTAGATATGAAATCACGCCATGTA

CACGGAAGGAATTACCCTGTTATCCCTATCTAGATATGAAATCACGCCATGTA

CACGGAAGGAATTACCCTGTTATCCCTATCTAGATATGAAATCACGCCATGTA

CACGGAAGGAATTACCCTGTTATCCCTATCTAGATATGAAATCACGCCATGTA

CACGGAAGGAATTACCCTGTTATCCCTATCTAGATATGAAATCACGCCATGTA

CACGGAAGGAATTACCCTGTTATCCCTATCTAGATATGAAATCACGCCATGTA

CACGGAAGGAATTACCCTGTTATCCCTATCTAGATATGAAATCACGCCATGTA

CACGGAAGGAATTACCCTGTTATCCCTATCTAGATATGAAATCACGCCATGTA

CACGGAAGGAATTACCCTGTTATCCCTATCTAGATATGAAATCACGCCATGTA

CACGGAAGGAATTACCCTGTTATCCCTATCTAGATATGAAATCACGCCATGTA

CACGGAAGGAATTACCCTGTTATCCCTATCTAGATATGAAATCACGCCATGTA

CACGGAAGGAATTACCCTGTTATCCCTATCTAGATATGAAATCACGCCATGTA

CACGGAAGGAATTACCCTGTTATCCCTATCTAGATATGAAATCACGCCATGTA

CACGGAAGGAATTACCCTGTTATCCCTATCTAGATATGAAATCACGCCATGTA

CACGGAAGGAATTACCCTGTTATCCCTATCTAGATATGAAATCACGCCATGTA

CACGGAAGGAATTACCCTGTT—CCCTATCTAGATATGAAATCACGCCATGTA

CACGGAAGGAATTACCCTGTT—CCCTATCTAGATATGAAATCACGCCATGTA

CACGGAAGGAATTACCCTGTT--CCCTATCTAGATATGAAATCACGCCATGTA

CACGGAAGGAATTACCCTGTT—CCCTATCTAGATATGAAATCACGCCATGTA

CACGGAAGGAATTACCCTGTT—CCCTATCTAGATATGAAATCACGCCATGTA

CACGGAAGGAATTACCCTG--ATCCCTATCTAGATATGAAATCACGCCATGTA

CACGGAAGGAATTACCCTG-TATCCCTATCTAGATATGAAATCACGCCATGTA

CACGGAAGGAATTACCCTG-TATCCCTATCTAGATATGAAATCACGCCATGTA

CACGGAAGGAATTACCCTG-TATCCCTATCTAGATATGAAATCACGCCATGTA

CACGGAAGGAATTACCCTG-TATCCCTATCTAGATATGAAATCACGCCATGTA

CACGGAAGGAATTACCCTG-TATCCCTATCTAGATATGAAATCACGCCATGTA

CACGGAAGGAATTACCCTGTTA-CCCTATCTAGATATGAAATCACGCCATGTA

CACGGAAGGAATTACCCTGTTA-CCCTATCTAGATATGAAATCACGCCATGTA

CACGGAAGGAATTACCCTGTTA-CCCTATCTAGATATGAAATCACGCCATGTA

CACGGAAGGAATTACCCTGTTA-CCCTATCTAGATATGAAATCACGCCATGTA

CACGGAAGGAATTACCCTGT---CCCTATCTAGATATGAAATCACGCCATGTA

CACGGAAGGAATTACCCTGT---CCCTATCTAGATATGAAATCACGCCATGTA

CAACACGGAAGGAATTACCCTGT-ATCCCGATCTAGATATGAAATCACGCCAT

Insertion  : 5 seq/ 191 total = 3%

GGAAGGAATTACCCTGTTAT-----ins TATCCCTAAT-----CCCTATCTAGATATGAAAT

CAACACGGAAGGAATTACCCTGT---ins AAT---TATCCCTATCTAGATATGAAATCACGCCATGT

CACGGAAGGAATTACCCTGT--ins T--TATCCCTATCTAGATATGAAATCAC

GGAATTACCCTGT------------------ins 141----------TATCCCTATCTAGATATGA

CACGGAAGGAATTACCCTGT--------------ins 177---------TATCCCTATCTAGATATGAAATC

Deletion  : 107 seq/ 191 total = 56%

CACGGAAGGAATTACCCTGTTATCC-del 1 mhom 1-TATCTAGATATGAAATCACGCCATG

CACGGAAGGAATTACCCTGTTATCC-del 1 mhom 1-TATCTAGATATGAAATCACGCCATG

GGCTAGCGCTCTAGA----del 112 mhom 2-------ATTCGAGCTCGCCCGGGGATCC

CACGGAAGGAATTACCCT-------del 9 mhom 4---ATCTAGATATGAAATCACGCCATGTAG

ACACGGAAGGAATTACCCT------del 9 mhom 4---ATCTAGATATGAAATCACGCCATG

ACACGGAAGGAATTACCCT------del 9 mhom 4---ATCTAGATATGAAATCACGCCATG

GCTGGCTAGCGCTCTAGA------del 173 mhom 4-------------GCAGAGTGAAGGAAGGA

CACGGAAGGAATTACCCT----del 9 mhom 4-----ATCTAGATATGAAATCACGCC

CACGGAAGGAAT------del 8 mhom 3 ------TATCCCTATCTAGATATGAAATCACGCCA

GCTGGCTAGCGCTCTAG-----del 51 mhom 1----------------CCATGTAGTGTATTGACC

CACGGAAGGAATTACCCTGTTATC-----del 6 mhom 4----TAGATATGAAATCACGCCATGTA

GTGGATAGCGGTTTGACT------------------del 275 mhom 2---------ATCTAGATATGAAATCA

CACGGAAGGAATTACCCTG-------del 14 mhom 1-------ATATGAAATCACGCCATGTAG

CACGGAAGGAATTACCCT------del 9 mhom 4-----ATCTAGATATGAAATCACGCCATG

AGACCCAAGCTGGCTAGCGCTCTAG-----del 211 mhom 0-------CCAACCAACAAGAGCT

CACGGAAGGAATTACCCT----del 9 mhom 4-----ATCTAGATATGAAATCACGCCATG

CACGGAAGGAATTACCCT------del 9 mhom 4----ATCTAGATATGAAATCACGCCATG

CTCTAGAGCAACACGGA---------del 28 mhom 2-------------------TATGAAATCACGCCATGTA

CACGGAAGGAAT----del 8 mhom 3----TATCCCTATCTAGATATGAAATCACGCCATG

CACGGAAGGAAT-------del 17 mhom 2----------CTAGATATGAAATCACGCCATGTA

CACGGAAGGAAT---------del 17 mhom 2--------CTAGATATGAAATCACGCCATGTA

CCCAAGCTGGCTAG-------------del 149 mhom 2----------TCGAGCCCTCATATACACA

TTGGCACCAAAATCAAC------------------del 203 mhom 0--------TATCCCTATCTAGATATG

ACACGGAAGGAAT------del 11 mhom 2-----CCCTATCTAGATATGAAATCAC

GCGCTCTAGAGCAACACGGA---------------del 28 mhom 2-------------TATGAAATCACGCCATGT

CACGGAAGGAATTACCCTGTT-----del 19 mhom 3--------------ATCACGCCATGTAGTGTATTG

CACGGAAGGAATTACCCTGTT---------del 19 mhom 3----------ATCACGCCATGTAGTGTATTG

GCTGGCTAGCGCTCTAGAGC------del 118 mhom 4------------TCGCCCGGGGATCC

CACGGAAGGAATTACCCT------del 9 mhom 4---ATCTAGATATGAAATCACGCCATGTAGTG

ACGACTCACTATAGGGAGACCCA--------del 67 mhom 2--------CGCCATGTAGTGTATTGAC

TACATCAATGGGCGTGGATA---------------del 301 mhom 0---------------CACGCCATGTAGTGTA

ACACGGAAGGAATTACCCTGT-----del 9 mhom 1----CTAGATATGAAATCACGCCATGTAGTG

CACGGAAGGAATTACCCTGTT------del 8 mhom 1---CTAGATATGAAATCACGCCATGTAG

AGAGCACACGGAAGGA-----------del219 mhom 5 -----------GACCACCATGTGCCGAGCCATCT

CACGGAAGGAATTACCCTG-------del 14 mhom 1-------ATATGAAATCACGCCATGTAGTGTAT

GAGCAACACGGAA----------del 21 mhom 1-----------TCTAGATATGAAATCACGCCAT

AGACCCAAGCTGGCTAGC------------del 36 mhom 1--------------CCTATCTAGATATGAAATCAC

CACGGAAGGAATTACCCTG-------del 19 mhom 2------------AAATCACGCCATGTAGTGTATTGA

CACGGAAGGAATTACCCTGTT—del 3 mhom 0-CCTATCTAGATATGAAATCACGCCATGT

CTCTAGAGCAACACGGAAGG------------del 331 mhom 6------------GGAATCAGCAGAACTGCCC

AAGGAATTACCCTGTTATC---del 6 mhom 4---TAGATATGAAATCACGCCATGTAG

GGAAGGAATTACCCTGT-------del 15 mhom 1--------ATGAAATCACGCCATGTAG

CACGGAAGGAAT----del 8 mhom 3----TATCCCTATCTAGATATGAAA

CACGGAAGGAATTACCCT----del 9 mhom 4-----ATCTAGATATGAAATCACGCCATGTAG

ACGGTGGGAGGTCTATATA------------------del 126 mhom 0--------------CCCTATCTAGATATGA

CACGGAAGGAAT-------del 17 mhom 2----------CTAGATATGAAATCACGCCATG

CACGGAAGGAAT------del 17 mhom 2-----------CTAGATATGAAATCACGCCATGTAGTGTA

CGGAAGGAATTACCCTGTTATC---del 6 mhom 4---TAGATATGAAATCACGCCATGTAGTG

AAGCTGGCTAGCGCTC-----del 38 mhom 6---------------TAGATATGAAATCACGCCATGTA

CACGGAAGGAAT-----------del 15 mhom 1----ATCTAGATATGAAATCACGCCAT

CACGGAAGGAATT-----------------del 77 mhom 4-------------ACCCGGTCACCCATTCGAAT

GCAACACGGAAGGAATTACCCT-----del 9 mhom 4----ATCTAGATATGAAATCACGCCATG

ACACGGAAGGAA-----------------del 172 mhom 6------------GGACTGGCCAGAGGC

CCAAGCTGGCTAGCGC ---------del 57 mhom 3---------CATGTAGTGTATTGACC

AGAGCAACACGGAAGGAATTA-------del 14 mhom 2-------TCTAGATATGAAATCACGCCATGTA

AGAGCAACACGGAAGGAATTA-----------del 22 mhom 2-----------TGAAATCACGCCATGTAGTGTAT

CTAGAGCAACACGGAAGGAAT----------del 25 mhom 2----------GAAATCACGCCATGTAGTG

CGGAAGGAATTACCCTGTTATC---del 6 mhom 3---TAGATATGAAATCACGCCATGTAGTGTAT

CTAGAGCAACACGGAAGGAAT------del 8 mhom 3--TATCCCTATCTAGATATGAAATCACGCCA

GGCTTATCGAAATTAATACG-------------del 138 mhom 2----------------GTCACCCATTCGAATTCGAG

CTCACTATAGGGAGACCCAAGC----------del 368 mhom 3------------------AGAACTGCCCTGCGAGA

CTAGAGCAACACGGAAGGAAT-----del 6 mhom 3---TATCCCTATCTAGATATGAAATCACG

TTGGCACCAAAATCAACGGGA--------del 212 mhom 2----------------TATGAAATCACGCCAT

TAACTAGAGAACCCACTGCT--------------------del 274 mhom 0------------CAACCAACAAGAGCTCAAGG

CACGGAAGGAATTACCCT-----del 9 mhom 4----ATCTAGATATGAAATCACGCCATGTA

GCTCTAGAGCAAACGGAA----------del 16 mhom 0------CCCTATCTAGATATGAAATCA

ACGGAAGGAATTACCCTGTTATC-----del 6 mhom 4---TAGATATGAAATCACGCCATGTAGTGTAT

GCGCTCTAGAGCAACACGGA-----------------del 203 mhom 2-------------ACCAACAAGAGCTCAAGG

ATAGGGAGACCCAAGCTGGC----------------del 262 mhom 2-------------CATCTCTCTTAGGCGCTTGCT

GGAGACCCAAGCTGGCTAGCGC----------del 57 mhom 3------------CATGTAGTGTATTGACCGATTCCT

TTATCGAAATTAATACGACTCACTAT-------------------del 64 mhom 4---------CTAGATATGAAATCACG

CGCTCTAGAGCAACACG------------------------del 42 mhom 4------------CCATGTAGTGTATTGACCGAT

TCTAGAGCAACACG--------------------del 42 mhom 4--------------CCATGTAGTGTATTGACCGATTCC

CTAGAGCAACACGGAAGGAAT------del 11 mhom 2-----CCCTATCTAGATATGAAATCACGCC

CACGGAAGGAATTACCCTGTTATC----del 6 mhom 3—TAGATATGAAATCACGCCATGTAGTGTATT

AACACGGAAGGAATTACCCT-----del 9 mhom 4----ATCTAGATATGAAATCACGCC

TCTAGAGCAACACG-----------------del 42 mhom 4--------------CCATGTAGTGTATTGACCGATT

GGCTAGCGCTCTAGAGCAACACG------------------del 42 mhom 4-----------CCATGTAGTGTATTGA

AACACGGAAGGAATTACCCT----9 mhom 4-----ATCTAGATATGAAATCACGCCATGTA

ATAGGGAGACCCAAGCT----------------del 58 mhom 0-------------AATCACGCCATGTAGTGTATT

ATCGAAATTAAT------------del 54 mhom 1-----------TATCCCTATCTAGATA

AAGTCTCCACCCC--------------del 248 mhom 3----------TATCTAGATATGAAATCACG

CACCCCATTGACGTCAA------------del 231 mhom 1---------TATCCCTATCTAGATATGAAA

CACGGAAGGAATTACCCT-------del 9 mhom 4—ATCTAGATATGAAATCACGCCATGTA

AGCGCTCTAGAGCAACAC--------del 23 mhom 1---------------TATCTAGATATGAAAT

AATTAATACGACTCACTAT---------------------del 64 mhom 4-------CTAGATATGAAATCACGCC

AGAGCAACACGGAAGGAATTACCCT------del 9 mhom 4---ATCTAGATATGAAATCACGCCATGTA

ACACGGAAGGAATTACCCTGT-----------------del 234 mhom 1--------CTTAGGCGCTTGCTGCTGCTG

TCTAGAGCAACACGGAAGG--------del 189 mhom 3-------------CTCAGATTCCCAACCAACAAGA

CTAGCGCTCTAGAGCAACAC-----------del 293 mhom 1---------------TAGCTGTCACTCAA

CTAGAGCAACACGGAAGGAAT----del 8 mhom 3----TATCCCTATCTAGATATGAAATC

AGCGCTCTAGAGCAAC----------------del 292 mhom 3------------TCCTAGCTGTCACTCAAGGGA

ACGGAAGGAATTACCCTGTTATC---del 6 mhom 4---TAGATATGAAATCACGCCATGTAGTGTA

TGGCTAACTAGAGAACC-----------------del 112 mhom 0---------AATCACGCCATGTAGTGTATT

CACGGAAGGAATTACCC-------------------del 328 mhom 1----------AGCAGAACTGCCCTGCGAG

ACGGAAGGAATTACCCTGTTAT-----del 12 mhom 2-------ATGAAATCACGCCATGTAGTGTA

ACACGGAAGGAA----------------del 172 mhom 6---------------GGACTGGCCAGAGGC

CTAGCGCTCTAGAGCAAC ---del 293 mhom 3------TCCTAGCTGTCACTCAAGGGAAGAC

GCTGGCTAGCGCTCTA-----------del 34 mhom 3----------TCTAGATATGAAATCACGCCATG

ACACGGAAGGAAT----del 8 mhom 3----TATCCCTATCTAGATATGAAATCACGCCATGT

AAAATGTCGTAACAACTCC---------------del 178 mhom 3-------------CTATCTAGATATGAAATCACGC

CAACACGGAAGGAATTACC-----del 10 mhom 2-----TATCTAGATATGAAATCACGCCA

CGGAAGGAATTACCCT-----del 9 mhom 4----ATCTAGATATGAAATCACGCC

GCTCTAGAGCAACACG-------------del 42 mhom 4----------CCATGTAGTGTATTGACCG

CACGGAAGGAATTACCCT-----del 9 mhom 4----ATCTAGATATGAAATCACGCCATGTA

GGAAGGAATTACCCTGTTATC----del 6 mhom 4—TAGATATGAAATCACGCCATGTAGTGT

CACGGAAGGAAT-------del 17 mhom 2----------CTAGATATGAAATCACGCCATGTAGTGT

Deletion / Insertion : 22 seq/ 191 total = 11%

CTAGAGCAACACGGAAGGA---del 16 / ins TA-------TATCTAGATATGAAATCACG

TAGAGCAACACGGAAGG----- del 15 / ins CG-----CCTATCTAGATATGAAAT

GGCGGTAGGCGTGTACGGTGGGA----del 136 / ins ATT-----CCTATCTAGATATGA

CTAGAGCAACACGGAA----del 24 / ins TGA---------AGATATGAAATCACGCCATGT

AAGCTGGCAAGCGCTATAGAGC----del 34 / ins AACACG------GATATGAAATCACGCCATGTA

CCCCATTGACGTCAATGGGA-------del 461 / ins ATTTGTG------------AGCAGAGTGAAGGA

CTCTAGAGCAACACGGAA----del 21 / ins TT-----TCTAGATATGAAATCA

CACGGAAGG------del 13 / ins GTAAT--------TCCCTATCTAGATATGAAA

ACTGGCTTATCGAAATTAATAC------del 85 / ins ACTA ---CACGCCATGTAGTGTATTGACCG

CCCAAGCTGGCTAGCGCTCTAGAGCAACAC -----del 270 / ins A--------GCTGCTGCAGCT

TAGAGCAACACGGAAGG-------del 15 / ins GTAATT-------CCTATCTAGATATGAAAT

TAGAGCAACACGGAAGGAATTA------del 8 / ins AT----TCCCTATCTAGATATGAAATC

GGCGTGTACGGTGGGAGGTC---------del 134 / ins A-------TATCTAGATATGAAATCA

GAGACCCAAGCTGGCTAGCGCT-----del 268 / ins TTAGAGCAACA------------CGCTTGCTGC

TTGACGTCAATGGGA------del 229 / ins ATTTGTTTTGGCA---------CCCTATCTAGATATGA

GGGCGTGGATAG--------del 279 / ins GTA-------ATCCCTATCTAGATATGA

CACGGAAGGAATTACCCTGTT-del 1 / ins GT—TCCCTATCTAGATATGAAATCACGCCATGTA

CACGGAAGGAATTACCCTGT--- del 11 / ins CCCGATCA----- AGATATGAAATCACGCCATGTA

CGGAAGGAATTACCCTGTT-------del 1 / ins 50--------TCCCTATCTAGATA

GAAGGAATTACCCT---del 5 / ins 145---------CCCTATCTAGATATGAA

GCTCTAGAGCAACACGGA---------del 17 / ins 42--------- CCCTATCTAGATATGA

TGGTGATGCGGTTTTGGCA------ del 708 / ins 156---------------------TGCT

**siBRCA2**

Use of 3’ overhangs : 33 seq/ 95 total = 35%

ACACGGAAGGAATTACCCTGTTATCCCTATCTAGATATGAAATCACGCCAT

ACACGGAAGGAATTACCCTGTTATCCCTATCTAGATATGAAATCACGCCAT

ACACGGAAGGAATTACCCTGTTATCCCTATCTAGATATGAAATCACGCCAT

ACACGGAAGGAATTACCCTGTTATCCCTATCTAGATATGAAATCACGCCAT

ACACGGAAGGAATTACCCTGTTATCCCTATCTAGATATGAAATCACGCCAT

ACACGGAAGGAATTACCCTGTTATCCCTATCTAGATATGAAATCACGCCAT

ACACGGAAGGAATTACCCTGTTATCCCTATCTAGATATGAAATCACGCCAT

ACACGGAAGGAATTACCCTGTTATCCCTATCTAGATATGAAATCACGCCAT

ACACGGAAGGAATTACCCTGTTATCCCTATCTAGATATGAAATCACGCCAT

ACACGGAAGGAATTACCCTGTTATCCCTATCTAGATATGAAATCACGCCAT

ACACGGAAGGAATTACCCTGTTATCCCTATCTAGATATGAAATCACGCCAT

ACACGGAAGGAATTACCCTGTTATCCCTATCTAGATATGAAATCACGCCAT

ACACGGAAGGAATTACCCTGTTATCCCTATCTAGATATGAAATCACGCCAT

ACACGGAAGGAATTACCCTGTTATCCCTATCTAGATATGAAATCACGCCAT

ACACGGAAGGAATTACCCTGTTATCCCTATCTAGATATGAAATCACGCCAT

ACACGGAAGGAATTACCCTGTTATCCCTATCTAGATATGAAATCACGCCAT

ACACGGAAGGAATTACCCTGTTATCCCTATCTAGATATGAAATCACGCCAT

ACACGGAAGGAATTACCCTGTTATCCCTATCTAGATATGAAATCACGCCAT

ACACGGAAGGAATTACCCTGTTATCCCTATCTAGATATGAAATCACGCCAT

ACACGGAAGGAATTACCCTGTTATCCCTATCTAGATATGAAATCACGCCAT

ACACGGAAGGAATTACCCTGTTATCCCTATCTAGATATGAAATCACGCCAT

ACACGGAAGGAATTACCCTGTTATCCCTATCTAGATATGAAATCACGCCAT

ACACGGAAGGAATTACCCTGTT--CCCTATCTAGATATGAAATCACGCCAT

ACACGGAAGGAATTACCCTGTT--CCCTATCTAGATATGAAATCACGCCAT

ACACGGAAGGAATTACCCTGTT--CCCTATCTAGATATGAAATCACGCCAT

ACACGGAAGGAATTACCCTGTT--CCCTATCTAGATATGAAATCACGCCAT

ACACGGAAGGAATTACCCTGTTA-CCCTATCTAGATATGAAATCACGCCAT

ACACGGAAGGAATTACCCTG-TATCCCTATCTAGATATGAAATCACGCCAT

ACACGGAAGGAATTACCCTG-TATCCCTATCTAGATATGAAATCACGCCAT

ACACGGAAGGAATTACCCTG-TATCCCTATCTAGATATGAAATCACGCCAT

ACACGGAAGGAATTACCCTG-TATCCCTATCTAGATATGAAATCACGCCAT

ACACGGAAGGAATTACCC---TATCCCTATCTAGATATGAAATCACGCC

ACACGGAAGGAATTACCCTG--ATCCCTATCTAGATATGAAATCACGCCATG

Insertion : 1 seq/ 95 total = 1%

ACGGAAGGAATTACCCTGTTAT----------------ins 161-------CCCTATCTAGATAT

Deletion : 46 seq/ 95 total = 48%

TCTAGAGCAACACGG----------------del 334 mhom 2-------GGAATCAGCAGAACTGCCCTGCG

CACGGAAGGAATTACCCTGTTATC---del 2 mhom 1—TATCTAGATATGAAATCACGCCATGTA

AGCGCTCTAGAGCAACACG-------------del 131 mhom 1--------AGTCGAGCCCTCATATACACACA

CTTTCCAAAATGTCGTAACA-------------del 202 mhom 2 ----------CGCCATGTAGTGTATTGACC

CACGGAAGGAATTACCCT------del 9 mhom 4----ATCTAGATATGAAATCACGCCATGTAGTGTA

CACGGAAGGAATTACCCT------del 9 mhom 4----ATCTAGATATGAAATCACGCCATGTAGTGTA

CACGGAAGGAATTACCCT------del 9 mhom 4----ATCTAGATATGAAATCACGCCATGTAGTGTA

CACGGAAGGAATTACCCT------del 9 mhom 4----ATCTAGATATGAAATCACGCCATGTAGTGTA

CACGGAAGGAATTACCCT------del 9 mhom 4----ATCTAGATATGAAATCACGCCATGTAGTGTA

CACGGAAGGAATTACCCT------del 9 mhom 4----ATCTAGATATGAAATCACGCCATGTAGTGTA

CACGGAAGGAATTACCCT------del 9 mhom 4----ATCTAGATATGAAATCACGCCATGTAGTGTA

CACGGAAGGAATTACCCT------del 9 mhom 4----ATCTAGATATGAAATCACGCCATGTAGTGTA

CACGGAAGGAATTACCCT------del 9 mhom 4----ATCTAGATATGAAATCACGCCATGTAGTGTA

CACGGAAGGAATTACCCT------del 9 mhom 4----ATCTAGATATGAAATCACGCCATGTAGTGTA

ACACGGAAGGAATTACC--------del 10 mhom 2---------TATCTAGATATGAAATCACGCCATGTA

CACGGAAGGAATTACCCTGTTATC---del 6 mhom 4---TAGATATGAAATCACGCCATG

CACGGAAGGAATTACCCT----------del 17 mhom 1-------ATGAAATCACGCCATGTAGT

AGCGCTCTAGAGCAACACGG---------del 42 mhom 0----------CATGTAGTGTATTGACCGATTC

GACCCAAGCTGGCTAGCGCTC--- -----del 386 mhom -----------TAGATATGAAATCACGCCATG

GCGCTCTAGAGCA ACACGG-----------del 342 mhom 1----------CAGAACTGCCCTGCGAGA

TGGCTAGCGCTC--------del 38 mhom 6--------------TAGATATGAAATCACGCC

ACACGGAAGGAATTACCCTGTTATC----del 6 mhom 4—TAGATATGAAATCACGCCATGTAG

TCTAGAGCAACACGGAAGG---------------del 331 mhom 6-----------GGAATCAGCAGAACTGCC

CACGGAAGGAATTACCC---del 6 mhom 0---CCCTATCTAGATATGAAATCACGCCA

CTGGCTAGCGCTCTAGAGCAACA----------del 287 mhom 3---------CAACTCCTAGCTGTCACTC

TGGCTAGCGCTCTAGAGCA-------del 41 mhom 2----------ATCACGCCATGTAGTGTATTGACC

CTCTAGAGCAACACGG------------------del 42 mhom 5----------CATGTAGTGTATTGACCGATTC

ACACGGAAGGAATTACCC---------del 13 mhom 2----TAGATATGAAATCACGCCATGTAGTG

CACGGAAGGAATTACCCT---del 9 mhom 4------ATCTAGATATGAAATCACGCCAT

AGCGCTCTAGAGCAACACGGAA----------------del 33 mhom 3-----ATCACGCCATGTAGTGTA

CCAAGCTGGCTAGCGCTC--------del 38 mhom 6----------------TAGATATGAAATCACGCCATGT

CTCTAGAGCAACACGGAA-------del 15 mhom 1--------TCCCTATCTAGATATGAAAT

AAGCTGGCTAGCGCTCTA--------del 34 mhom 3-------------TCTAGATATGAAATCACGCCATGT

CACGGAAGGAATTACCCT---del 5 mhom 1----CCCTATCTAGATATGAAATCACGCCATG

CACGGAAGGAATTA-----del 14 mhom 2---------TCTAGATATGAAATCACGCCA

GCGCTCTAGAGCAACACGG-------del 23 mhom 0----------------TCTAGATATGAAATCACG

CACGGAAGGAAT---------------del 38 mhom 2------GTAGTGTATTGACCGATTCCTTGCGG

CACGGAAGGAATTACCCT------del 23 mhom 0-----------------TCACGCCATGTAGTGTATTG

CTAGCGCTCTAGAGCAACAC-----------del 303 mhom 3----------------TCAAGGGAAGACGCTGG

CACGGAAGGAA------------del 29 mhom 3-----------------ATCACGCCATGTAGTGTATTGACCGA

CACGGAAGGA--------del 24 mhom 2----------------TATGAAATCACGCCATGTAGTGTATTGA

CACGGAAGGAATTACCCTGTTATCC---del 1 mhom 1---TATCTAGATATGAAATCACGCCATG

CACGGAAGGAATTACCCT---del 5 mhom 1—CCCTATCTAGATATGAAATCACGCC

CACGGAAGGAATTACCCTGTTA------del 17 mhom 1-----------AATCACGCCATGTAGTGTATT

GAGCTCTCTGGCTAACTA---------------del 108 mhom 3--------TCTAGATATGAAATCACGCCA

GACCCAAGCTGGCTAGCGCTC--------del 304 mhom 3----CTAGCTGTCACTCAAGGGAAG

Deletion / Insertion : 15 seq/ 95 total = 16%

CTCTAGAGCAACACGGAA----del 19 / ins TTCCTTCA-------- TATCTAGATATGAAATCAC

TAGCGCTCTAGAGCAACAC-----------del. 17 / ins CA---TATCCCTATCTAGATATGAA

CTCTAGAGCAACACG---------------del 312 / ins C-------------ACGCTGGTGCTGGGGAAGGAAG

CACGGAAGGAATTAC------del 11 / ins A----TATCTAGATATGAAATCACGCCATGTAGT

TGGATAGCGGTTTGACTCAC--------del 291 / ins C----------CCATGTAGTGTATTGACCGATTC

GCAACACGGAAGGAATTACCCT---------del 6 / ins AGGAATTT-------CCTATCTAGATATGAAATCAC

TAGGGAGACCCAAG------del 329 / ins G---AAGGGAAGACGCTGGTGCTGGGGAA

CACGGAAGGAATTACCC----del 11 / ins AG----TCTAGATATGAAATCACGCCATGTAGT

GAGACCCAAGCTGGCTAG---del 309 / ins TAG--------TAGCTGTCACTCAAGGGAAG

ACCCCATTGACGTCAATGGG-G------del 302 / ins GTGAC------GTCACCCATTCGAATTCGAGCT

ACACGGAAGGAATTACCCTGT--del 4 / ins AATT-----CCTATCTAGATATGAAATCACGCCAT

GCTCTAGAGCAACACGGAAGGAAT------del 14 / ins 110-------TATCTAGATATGAAATCACG

CCGCCCCATTGACGCAAC -----------------del 242 / ins 164--------TTCGAATTCGAGCTCGCCC

CACGGAAGGAATTAC------del 11 / ins AG----TATCTAGATATGAAATCACGCCATGTAGT

TATAGGGAGACCCAAGC-----del 48 / ins AGGCTAGCGC----TCTAGATATGAAATCACGCCATGTAGT

**Supplementary data S4**

**Supplementary data S4A. Impact of the siRNA on the cell cycle distribution.**


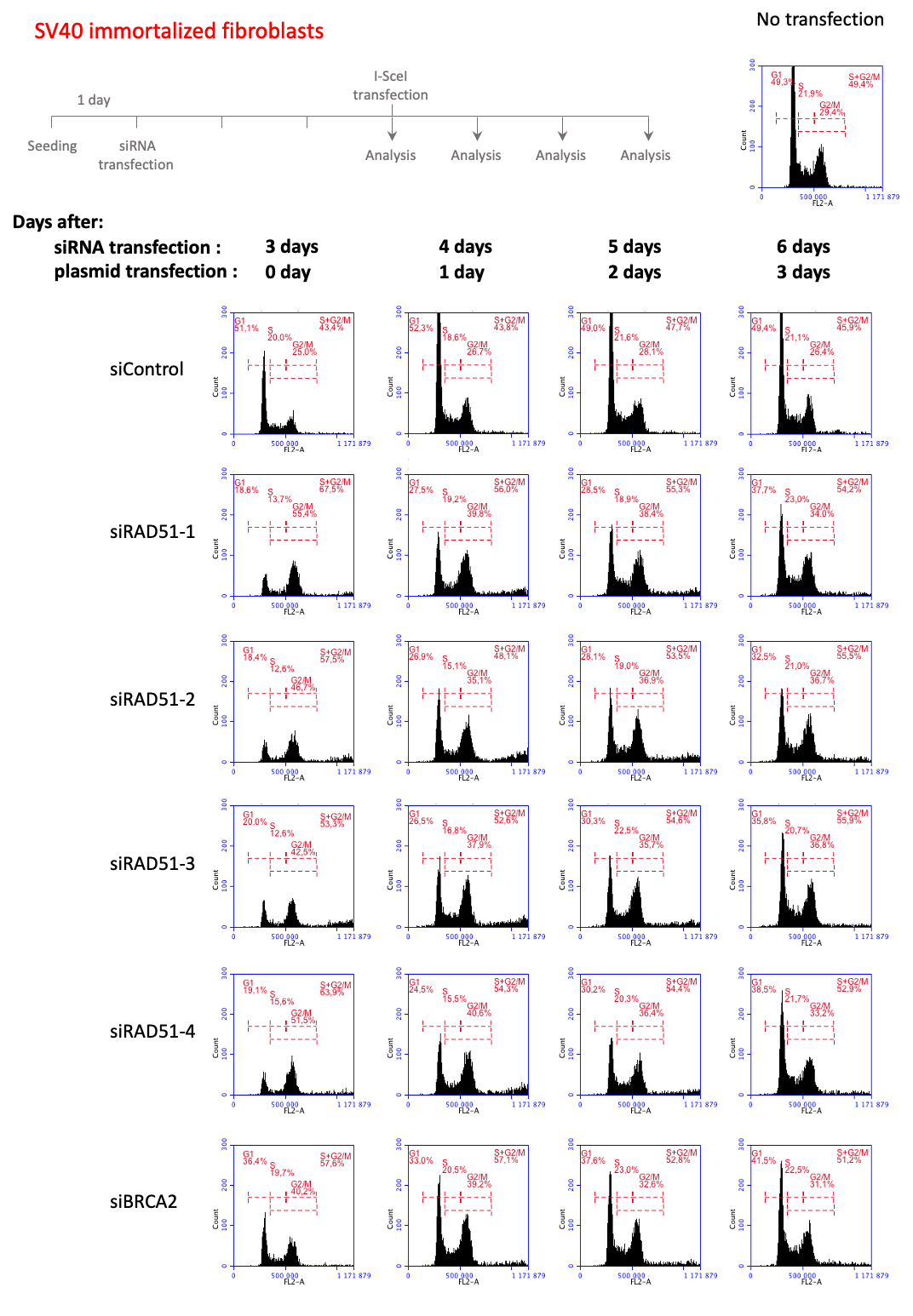
 **
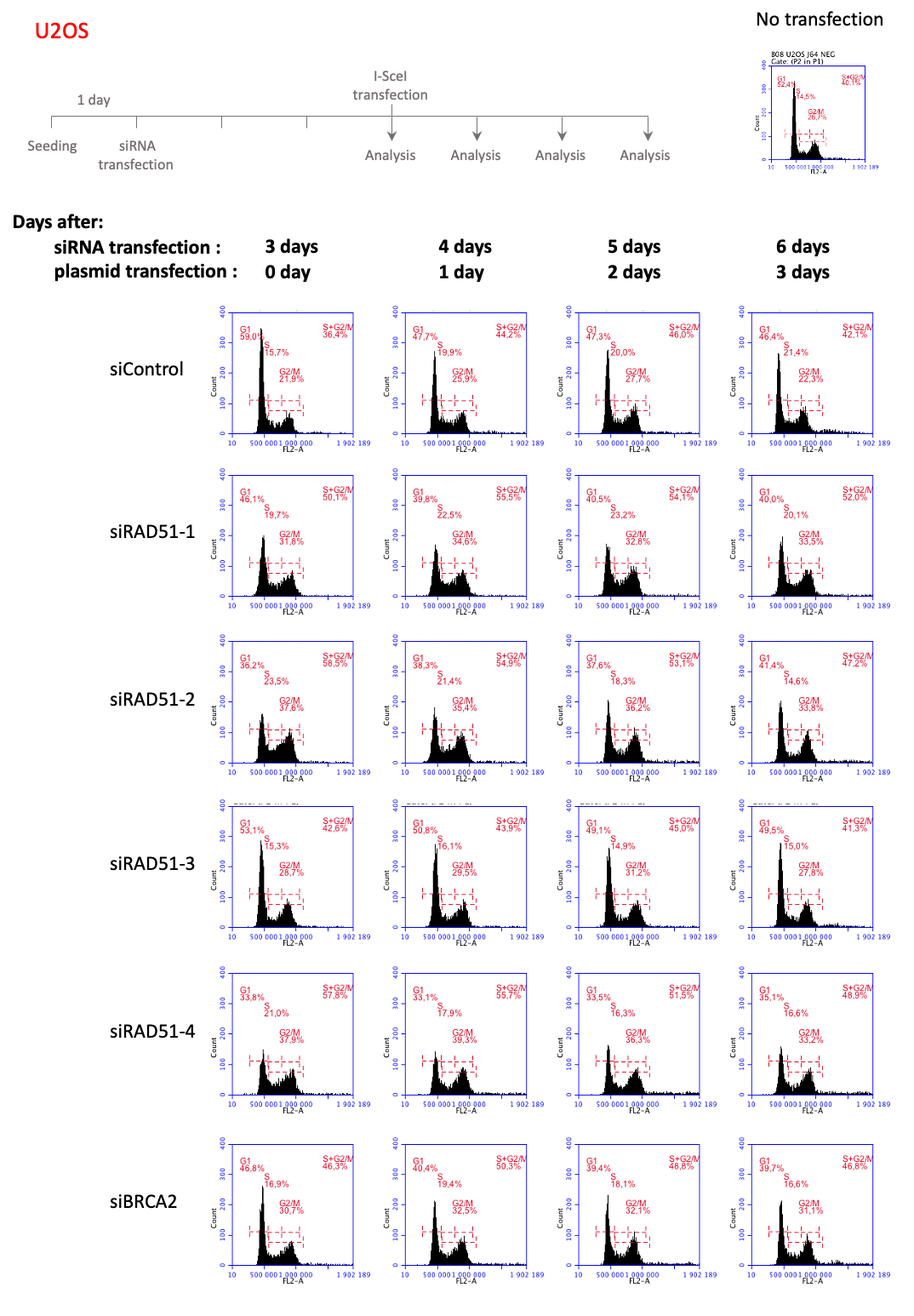
**

Counts

DNA content (PI staining)

**Supplementary data S4B. Impact of the DN-RAD51 on the cell cycle distribution.**

**Supplementary Figure S4. Cell cycle distribution of SV40-transformed fibroblasts or U2OS cells, transfected with siRNA against RAD51 or BRCA2 or expression plasmids for WT- or DN-RAD51s. A.** Cell cycle distribution of SV40-transformed fibroblasts and U2OS cells transfected with siRNA targeting RAD51 or BRCA2 (+I-SceI transfection). **B.** Cell cycle distribution of SV40-transformed fibroblasts transfected with plasmids coding for the different DN-RAD51 + I-SceI.

**Methods :**

Cells were detached, pelleted and fixed in ice-cold 70% ethanol. After an o/n incubation at -20°C, cells were pelleted, washed in PBS and incubated in PBS containing propidium iodide (5µg/mL, Sigma Aldrich) and RNase A (50µg/mL, Sigma Aldrich). PI content was scored by FACS analysis using a BD Accuri C6 flow cytometer (BD biosciences).

**Supplementary data S5**: **Dose response of radiation induced RAD51 foci.**

The plateau of radiation-induced foci was reached at 6Gy.

**Supplementary Figure S5. Dose-dependent induction of RAD51 foci in GC92 cells** expressing only endogenous RAD51 (cells transfected with an empty plasmid, black bars) or cells over-expressing exogenous WTRAD51 (green bars) or the dominant negative SMRAD51 (red bars). Cells were irradiated with 3, 6 and 9 Gy of γ-rays.

Immunofluorescence and foci counting were performed as described in the Material and Methods section of the article.

Bars represent the mean ± SEM of 3 to 4 individual experiments.

**Supplementary data S6**


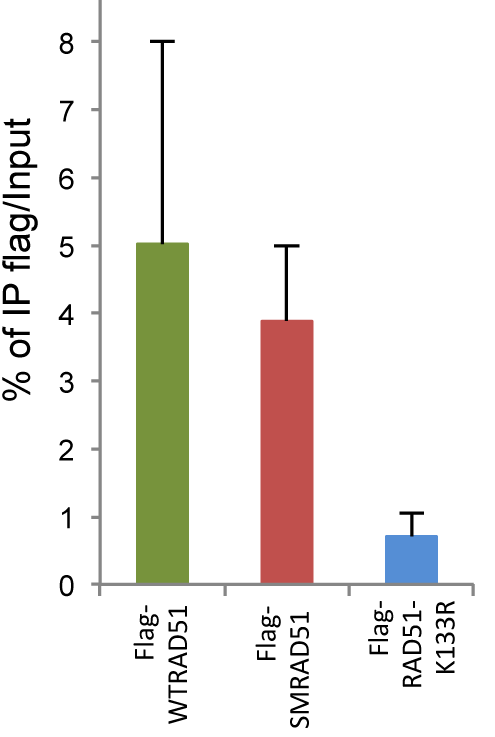


**Supplementary Figure S6. ChIP for Flag-RAD51s on Asi-SI induced DSBs in DIvA cells.**

We used the DIvA system (1–3), in which DSBs are generated upon the nuclear translocation of the restriction endonuclease Asi-SI. Chromatin immunoprecipitation (ChIP) was performed with anti-Flag antibody, and the DNA sequences bound to Flag-RAD51 were quantified by qPCR using specific primers surrounding the cleavage sites. The data showed that SMRAD51 bound to the cleaved DNA sites as efficiently as the wild-type WTRAD51. In contrast, RAD51-K133R, poorly bound to the cleaved DNA.

**Methods:**

DIvA cells (25 × 10^6^) were transfected by electroporation (Amaxa Cell Line Nucleofector kit V, Lonza Biosciences) using 20 µg of plasmid coding for the different forms of RAD51. Cells were seeded in a plate; 24 h later, the cells were treated or not with 4-hydroxy-tamoxifen (300 nM final concentration) for 4 h. The cells were crosslinked with formaldehyde (1%, 20 min) followed by cell lysis, DNA sonication and immunoprecipitation of the protein/DNA complexes with specific antibodies (anti-Flag M2, Sigma) or without antibodies as a negative control. DNA/protein complexes were collected with a mix of protein G and A agarose beads. Crosslinking was reversed by the addition of NaCl and then samples were treated with RNase A and proteinase K and DNA purified by centrifugation using a GFX PCR column (Amersham). DNA was quantified by qPCR using the SYBR Green qPCR master mix (Biorad) and the following specific primers: (1) DSB site, 5'-GATTGGCTATGGGTGTGGAC-3'and 5'-CATCCTTGCAAACCAGTCCT-3' and (2) control, 5'-GGCGACCTGGAAGTCCAACT -3' and 5'- CCATCAGCACCACAGCCTTC -3'.

**References**

1. Iacovoni,J.S., Caron,P., Lassadi,I., Nicolas,E., Massip,L., Trouche,D. and Legube,G. (2010) High-resolution profiling of cH2AX around DNA double strand breaks in the mammalian genome. *EMBO J.*, **29**, 1–12.

2. Caron,P., Aymard,F., Iacovoni,J.S., Briois,S., Canitrot,Y., Bugler,B., Massip,L., Losada,A. and Legube,G. (2012) Cohesin protects genes against γH2AX Induced by DNA double-strand breaks. *PLoS Genet.*, **8**, e1002460.

3. Aymard,F., Bugler,B., Schmidt,C.K., Guillou,E., Caron,P., Briois,S., Iacovoni,J.S., Daburon,V., Miller,K.M., Jackson,S.P., *et al.* (2014) Transcriptionally active chromatin recruits homologous recombination at DNA double-strand breaks. *Nat. Struct. Mol. Biol.*, **21**, 366–374.

**Supplementary data S7**

For irradiation in G1 and S phases, cells were blocked in late G1 with mimosine (Supplementary Figure S7 A and B). BrdU incorporation shows that cells did not synthetize DNA (Supplementary Figure S7 C). Then mimosine was either maintained for irradiation and until sample collection for foci analysis (G1), or mimosine was removed and cells were released for two additional hours before irradiation (Supplementary Figure S7A). Two hours after release, BrdU incorporation restarted and cells were in early S phase (S). In both cases, radiation-induced foci were analysed 6 hours after irradiation so that cells irradiated in S-phase (2 hours after mimosine release) did not reach the G2 phase at time of sample collection and foci analysis (see Supplementary Figure S7B). For irradiation in G2, cells were blocked with RO3306 (Supplementary Figure S7B), irradiated and analysed 6 hours after irradiation, maintaining the block.

In each cell cycle phases, the frequency of RAD51 foci was not significantly different when over expressing WTRAD51, SMRAD51 or no exogenous RAD51 (Supplementary Figure S7D).

**Supplementary Figure S7. Analysis of RAD51 foci in cells synchronized in G1, S or G2 phase of the cell cycle. A.** Experimental scheme. Foci were analysed 6 hours after 6Gy irradiation. G1 phase: Cells were treated o/n with 300μM of mimosine (Sigma Aldrich) and then irradiated. Mimosine was maintained in the medium until cells collection for foci analysis 6 hours after irradiation. S phase: Cells were treated with 300 μM of mimosine o/n and then released for 2 hours before irradiation. Cells were then left with no drug until cells collection, 6 hours after irradiation. G2 phase: Cells were synchronized with RO3306 (7 μM, Calbiochem) o/n. RO3306 was maintained in the medium during irradiation and until cells collection, 6hours after irradiation. **B.** Cell cycle profile of asynchronous, mimosine- and RO3306- treated cells. Histograms show cell count function of DNA content (PI staining). **C.** BrdU incorporation in cells treated with mimosine with or without a 2 hour-release. BrdU incorporation (10μM for 15 min, Sigma Aldrich) shows that cells mostly entered S-phase when treated with mimosine o/n and released for 2 hours with no drug. **D.** RAD51 foci in cells irradiated in G1, S or G2 phases of the cell cycle. Bars represent the mean ± SEM of 2 to 3 individual experiments in GC92 cells transfected with an empty plasmid or plasmids coding for WTRAD51 or SMRAD51.

**Supplementary data S8**

**Supplementary Figure S8. Impact of PARI and FBH1 on GC and SSA. A.** Impact of PARI on GC and SSA. Left panel**:** Western blot showing the expression of PARI, HA-I-SceI, and RAD51 in RG37 cells**.** Middle and right panel**:** impact of PARI on GC (middle panel, RG37 cells) and SSA (right panel, U2OS SSA cells) transfected with an empty plasmid or plasmids coding for WTRAD51 and RAD51-K133A. **B.** Impact of FBH1 on GC and SSA. Left panel**:** Western blot showing the expression of HA-FBH1 (detected with an antibody directed against the HA tag), HA-I-SceI, and RAD51 in RG37 cells**.** Middle and right panel**:** impact of FBH1 on GC (middle panel, RG37 cells) and SSA (right panel, U2OS SSA cells) transfected with an empty plasmid or plasmids coding for WTRAD51 and RAD51-K133A. In both cell lines and for both PARI and FBH1, histograms represent the mean ± SEM of 8 to 10 individual experiments.
